# Supplementary material for: Synthesis and Structure–Activity Relationship Studies of Pyrido [1,2-e]Purine-2,4(1H,3H)-Dione Derivatives Targeting Flavin-Dependent Thymidylate Synthase in Mycobacterium tuberculosis
Source: Molecules. 2022 Sep 21;27(19):6216. doi: 10.3390/molecules27196216 (PMC9571937; doi:10.3390/molecules27196216)

Synthesis and structure–activity relationship studies of pyrido[1,2-*e*]purine-2,4(*1H,3H*)-dione derivatives targeting *flavin-dependent thymidylate synthase* in *Mycobacterium tuberculosis*

Nicolas G. Biteau,[1] Vincent Roy,[1,*] Cyril Nicolas,[1] Hubert F. Becker,[2,3] Jean-Christophe Lambry,[2] Hannu Myllykallio,[2] and Luigi A. Agrofoglio[1,*]

[1] Institute of Organic and Analytical Chemistry, CNRS UMR 7311, Université d'Orléans, Rue de Chartres, 45067 Orleans CEDEX 2, France
[2] Laboratory of Optics and Biosciences, INSERM U 696-CNRS UMR 7645, Ecole Polytechnique, Route de Saclay, 91128 Palaiseau Cedex (France)
[3] Sorbonne Université, Faculté des Sciences et Ingénierie, 75005 Paris, France

Corresponding authors : vincent.roy@univ-orleans.fr; luigi.agrofoglio@univ-orleans.fr

Supplementary Informations

Copies of ^{1}H-, ^{13}C- and ^{1}H-^{13}C HMBC NMR spectra of representative molecules

- ✓ NMR analysis of representative compounds from isocyanate cyclisation compounds (**9c, 9e, 9g-j, 10b-d**)
- ✓ For the series **13, 18, 19, 20** and **21**, we present copies of ^{1}H-, ^{13}C-NMR spectra of representative molecules which exibit Mtb Thy X inhibition > 35%

^{1}H NMR (DMSO-d6) of compound **9c**

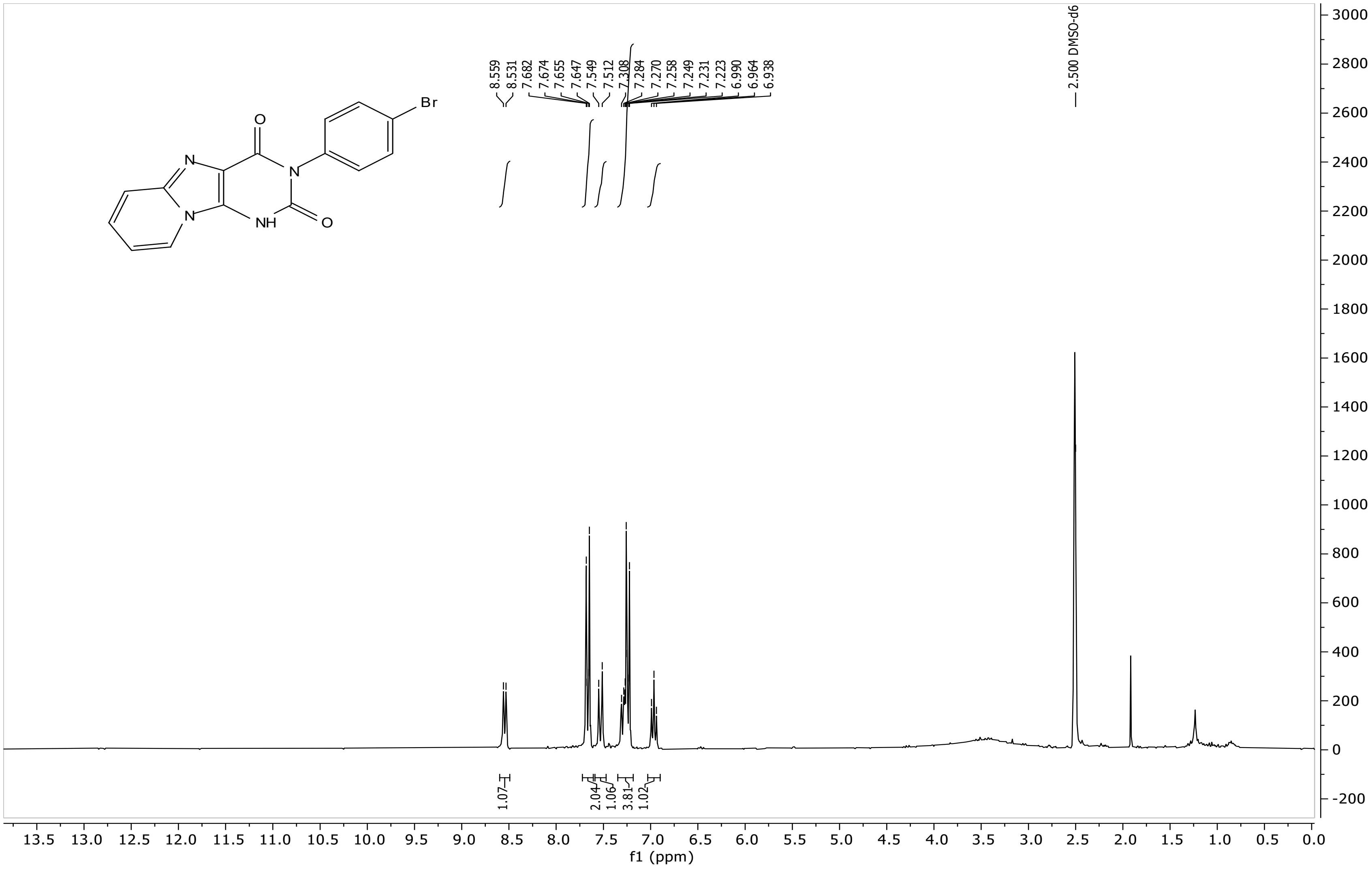

^{13}C NMR (DMSO-d6) of compound **9c**

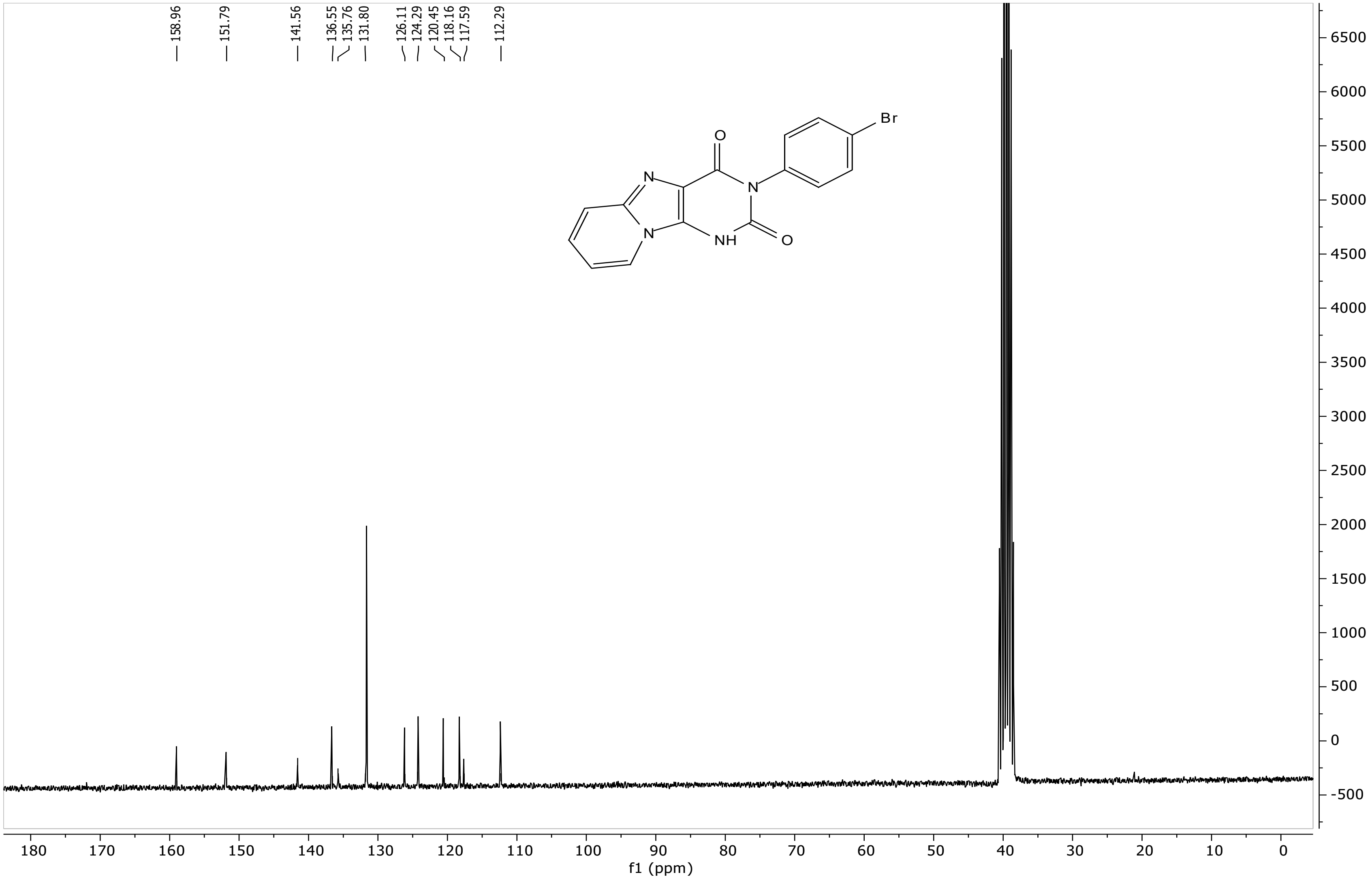

^{1}H NMR (DMSO-d6) spectra of compound **9e**

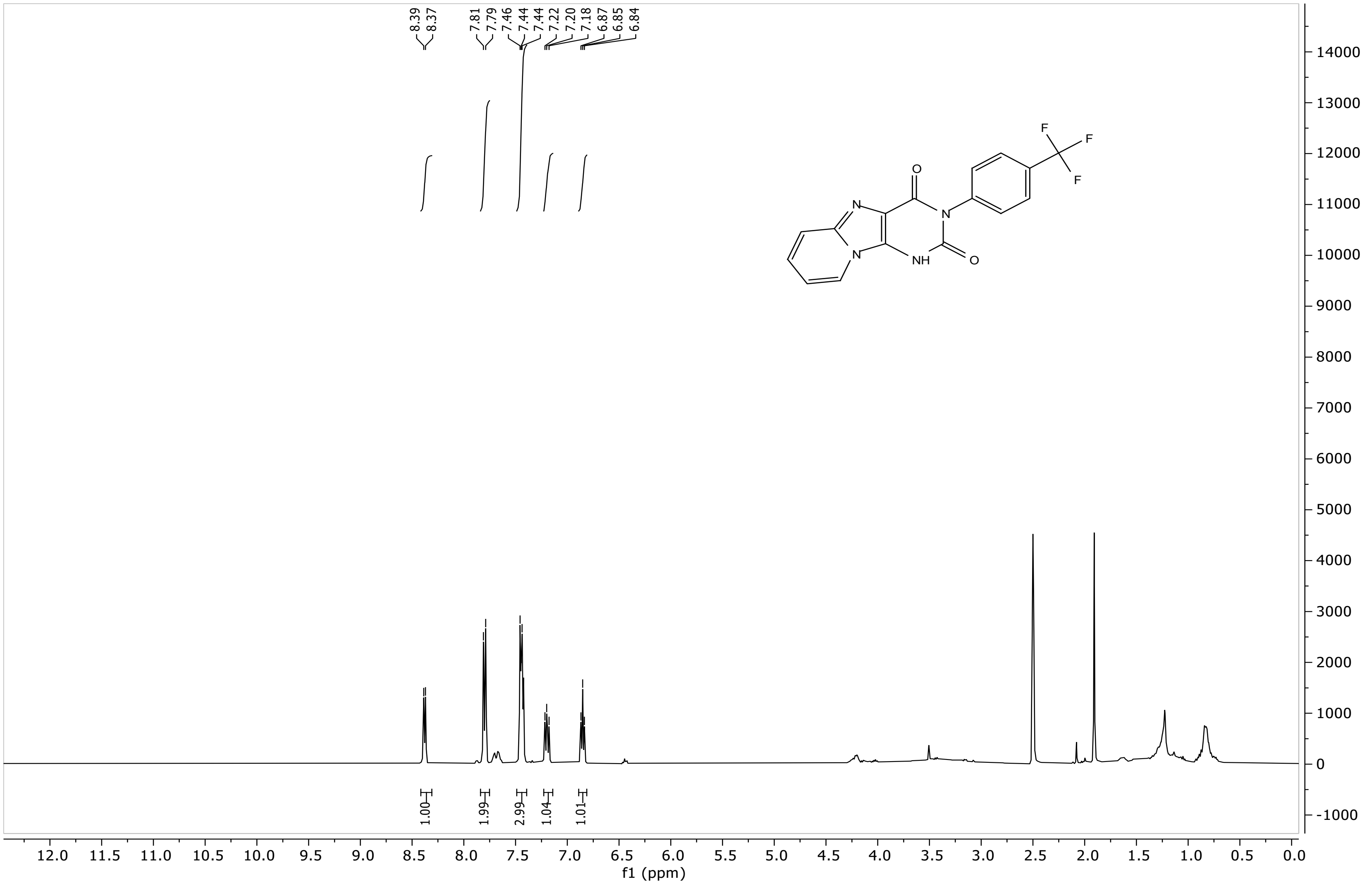

^{13}C NMR (DMSO-d6) spectra of compound **9e**

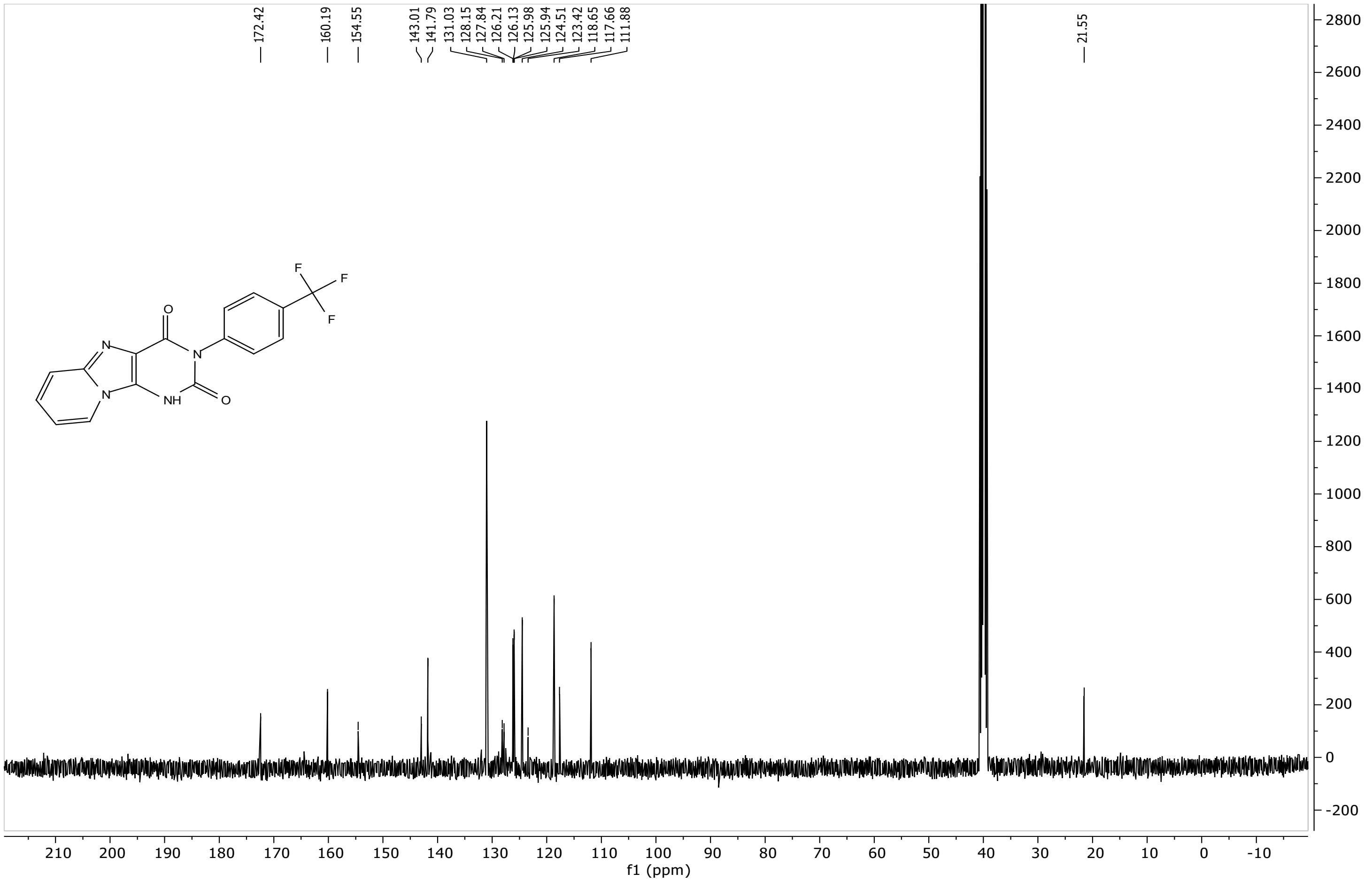

^{1}H NMR (DMSO-d$_6$) spectra of compound **9g**

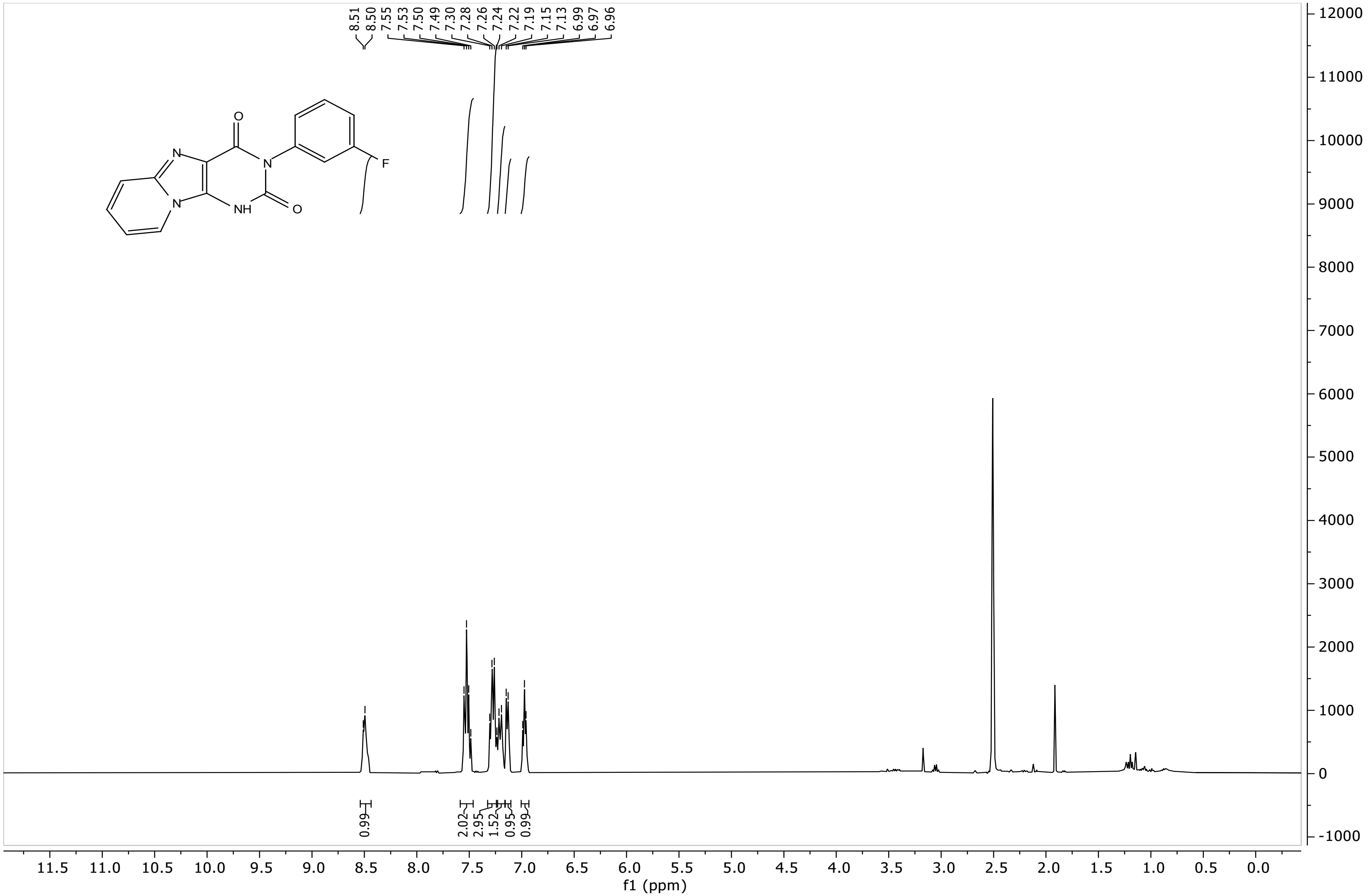

^{13}C NMR (DMSO-d_6) spectra of compound **9g**

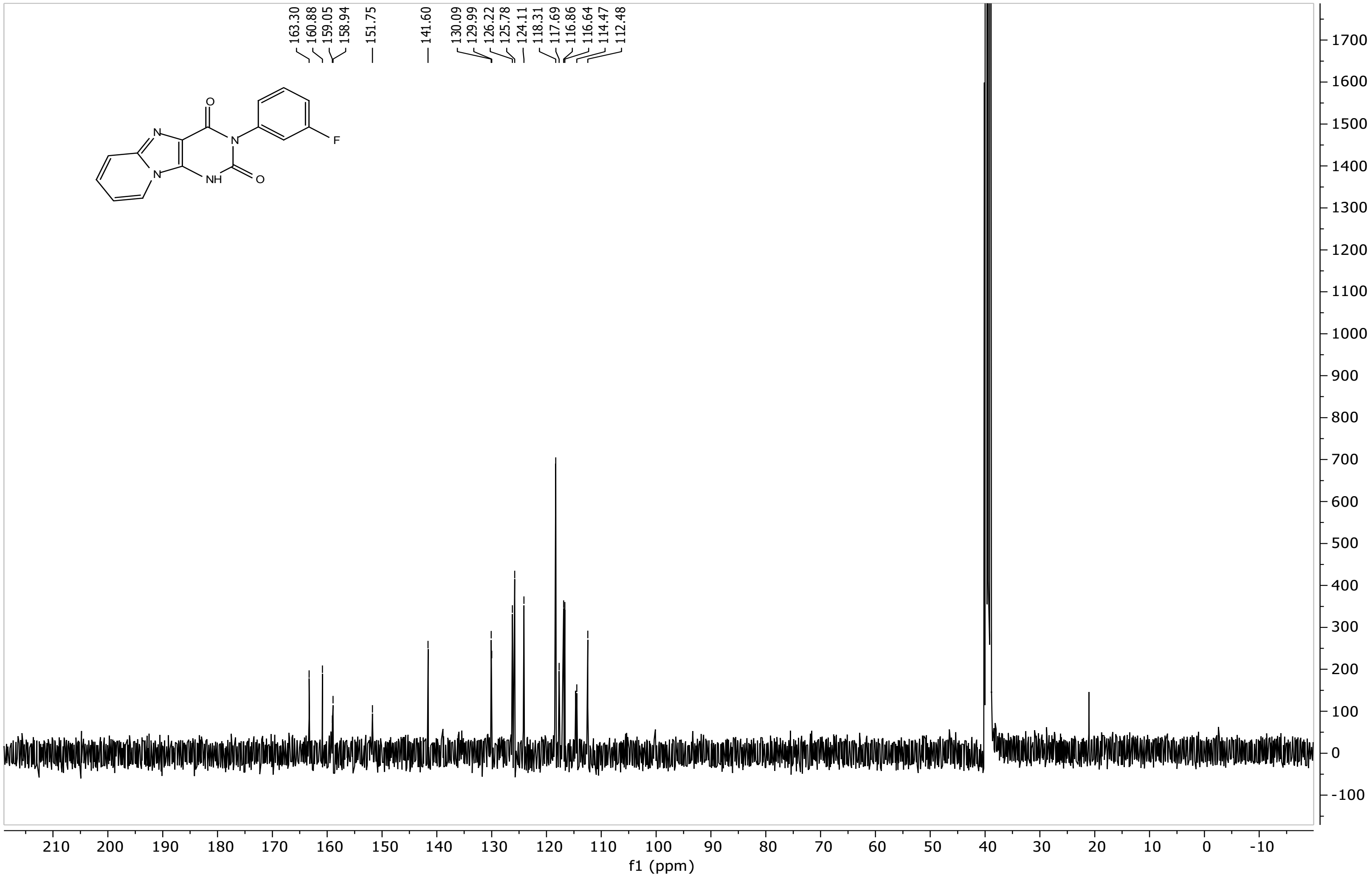

^{1}H NMR (DMSO-d$_6$) spectra of compound **9h**

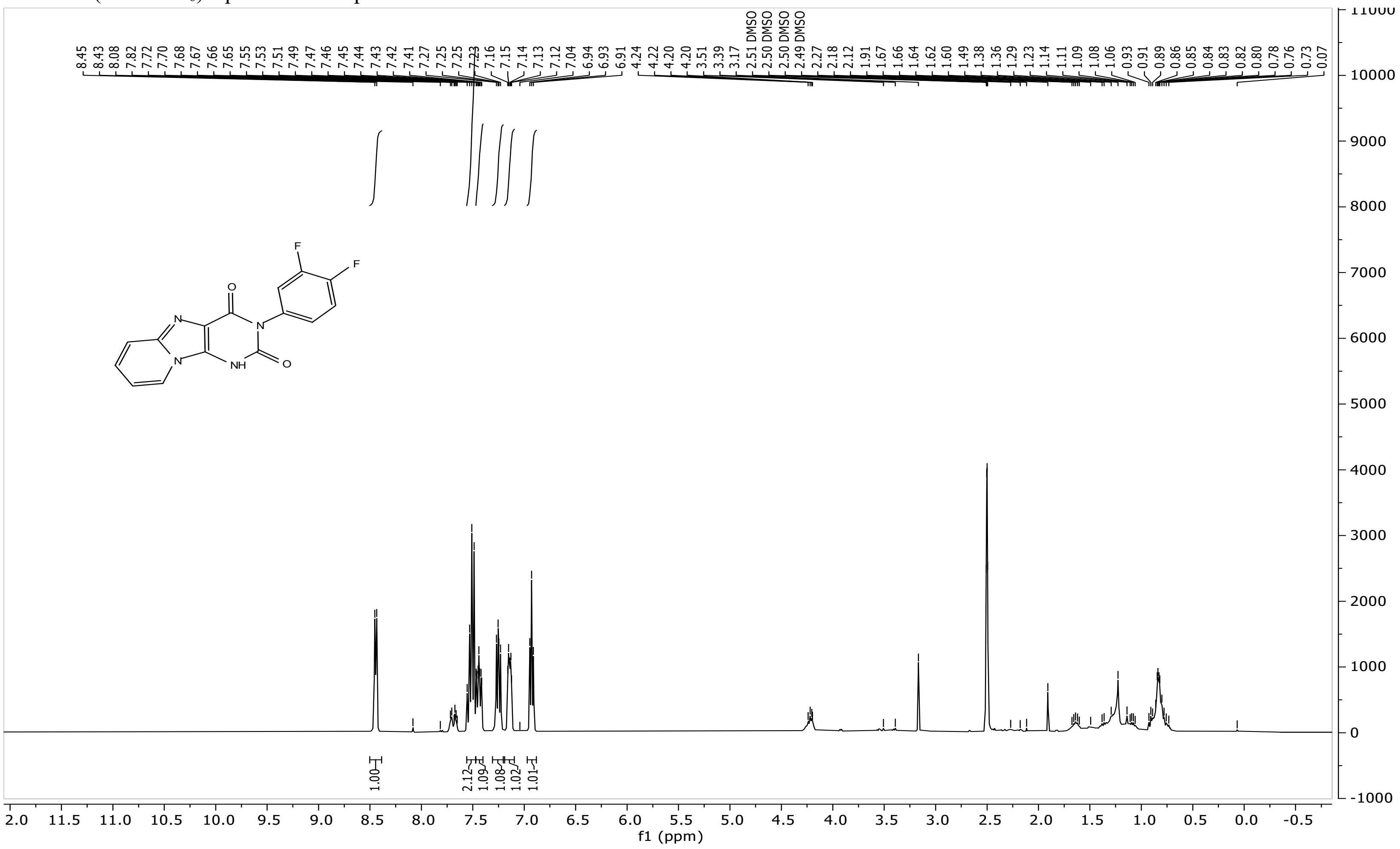

^{13}C NMR (DMSO-d6) spectra of compound **9h**

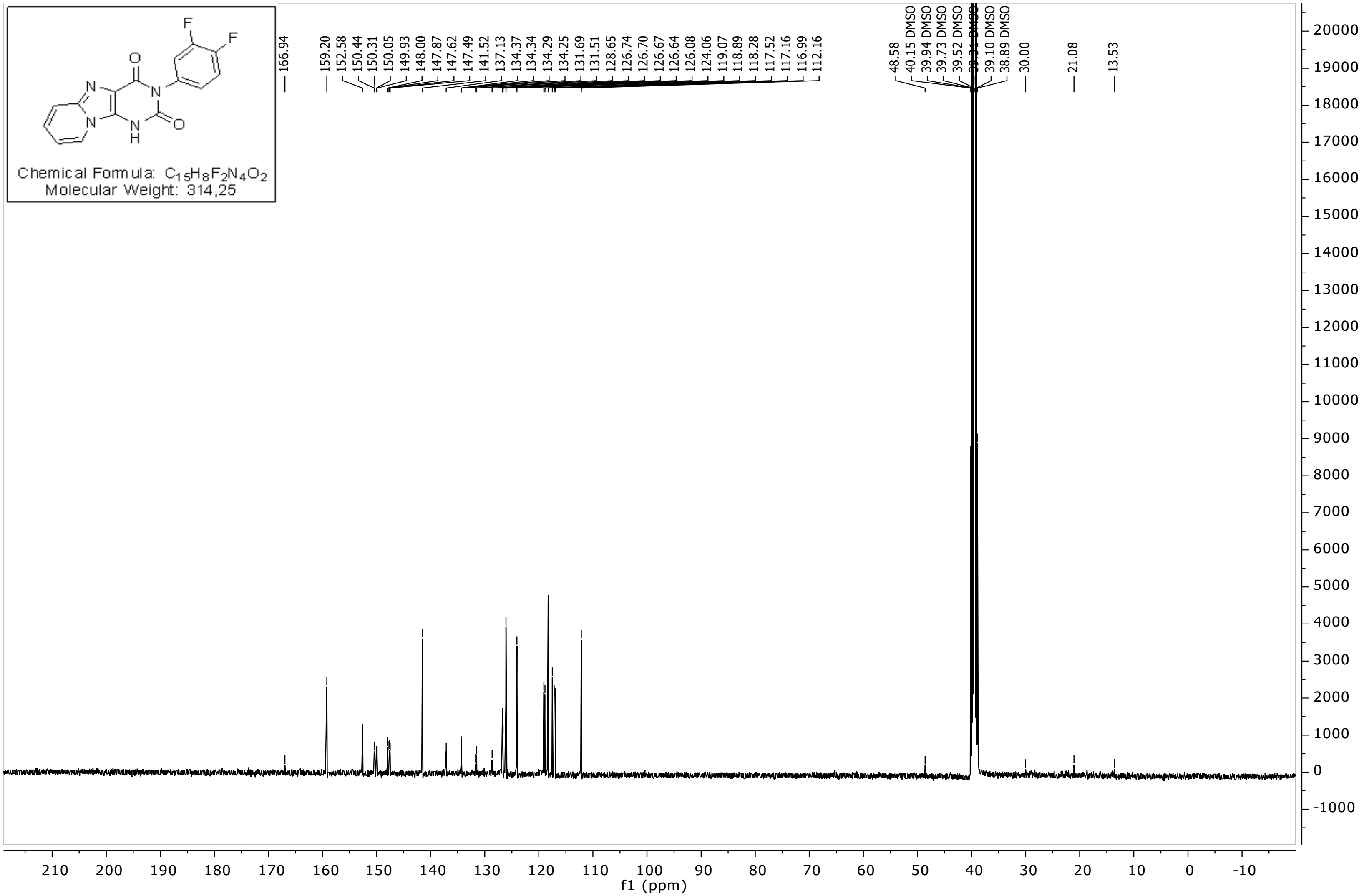

^{1}H and (DMSO-d$_6$) spectra of compound **9i**

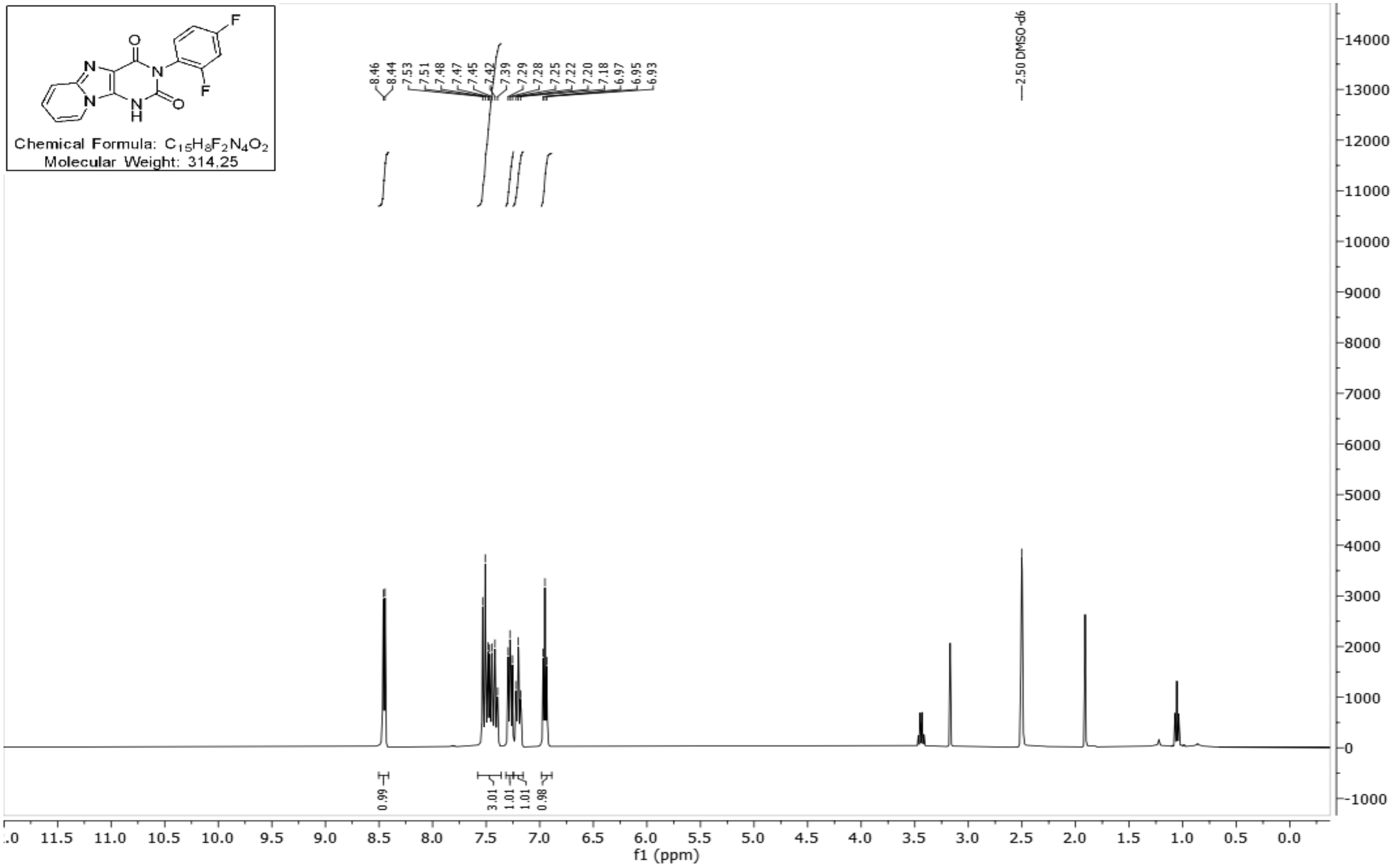

^{13}C NMR (DMSO-d6) of compound **9i**

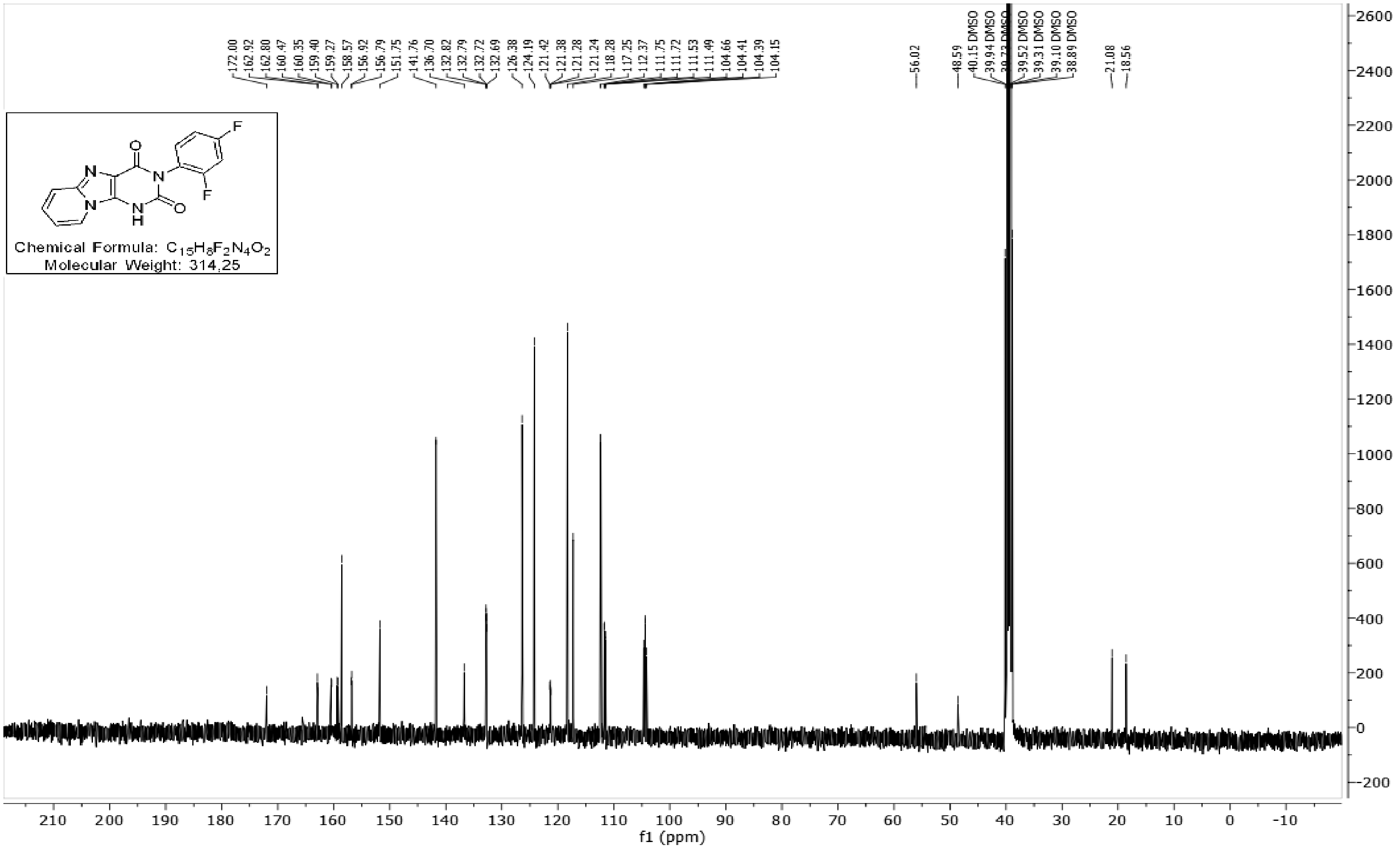

^{1}H NMR (DMSO-d6) of compound **9j**

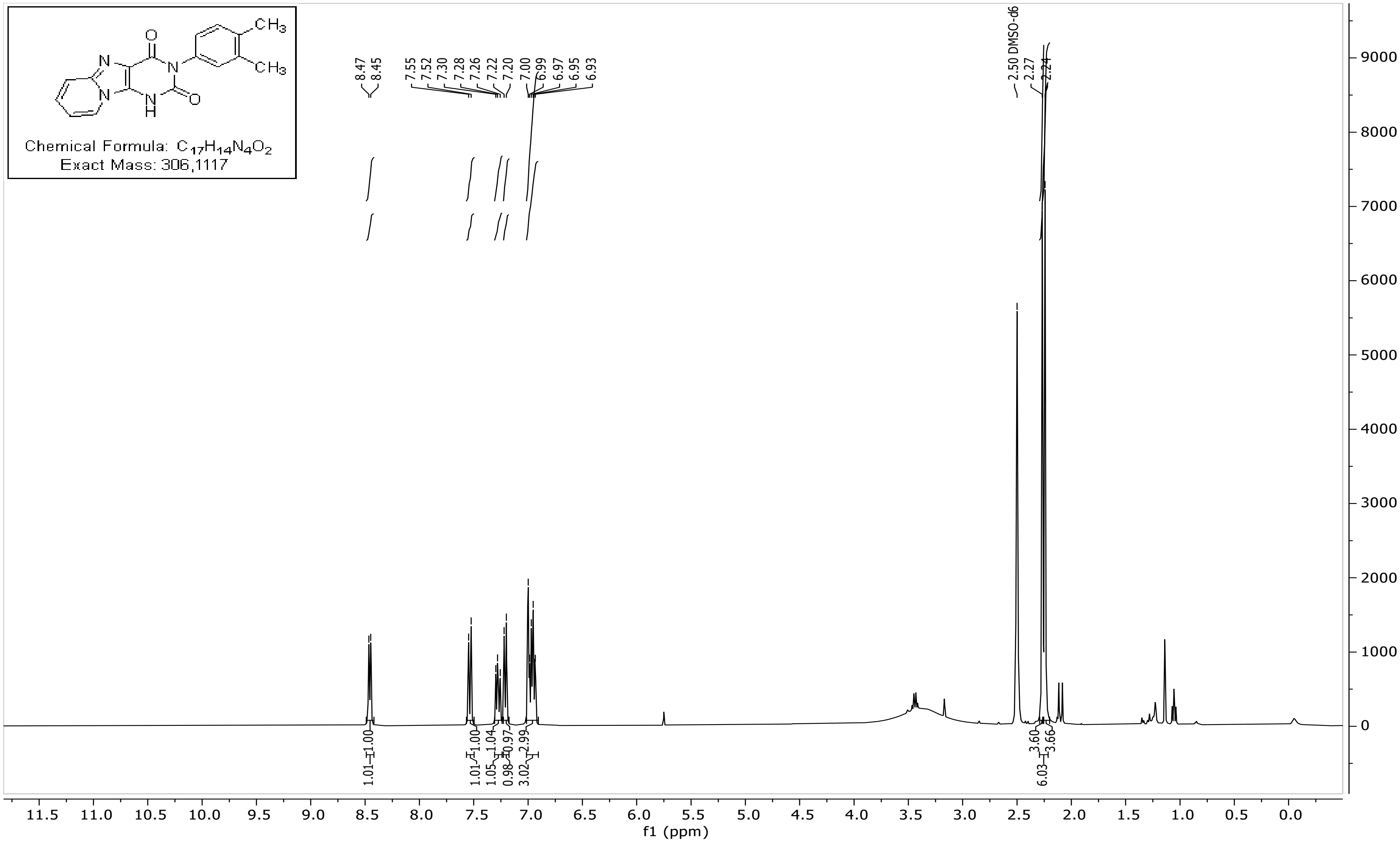

^{13}C NMR (DMSO-d6) of compound **3a**

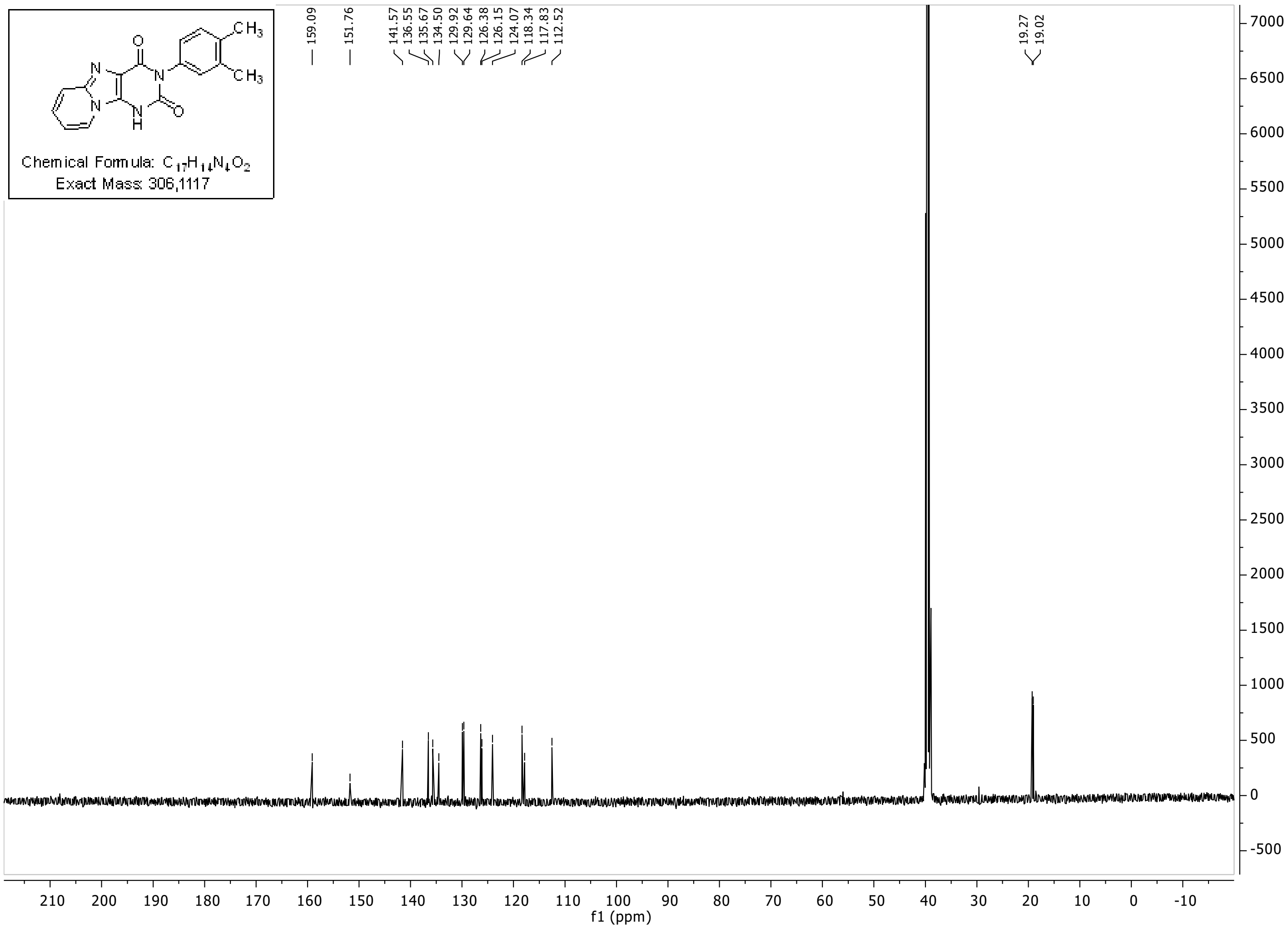

^{1}H NMR (DMSO-d6) of compound **10b**

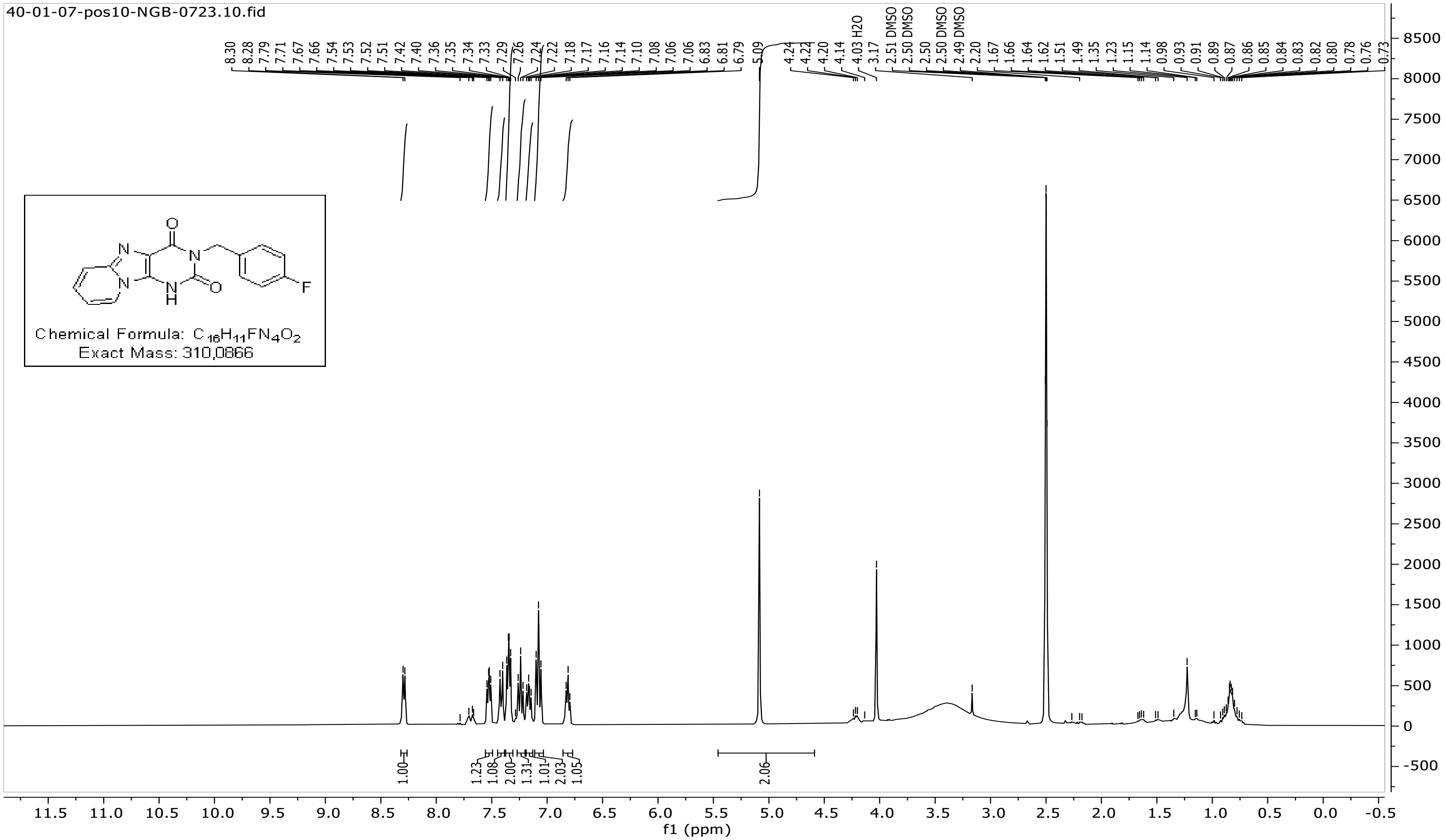

^{13}C NMR (DMSO-d6) of compound **10b**

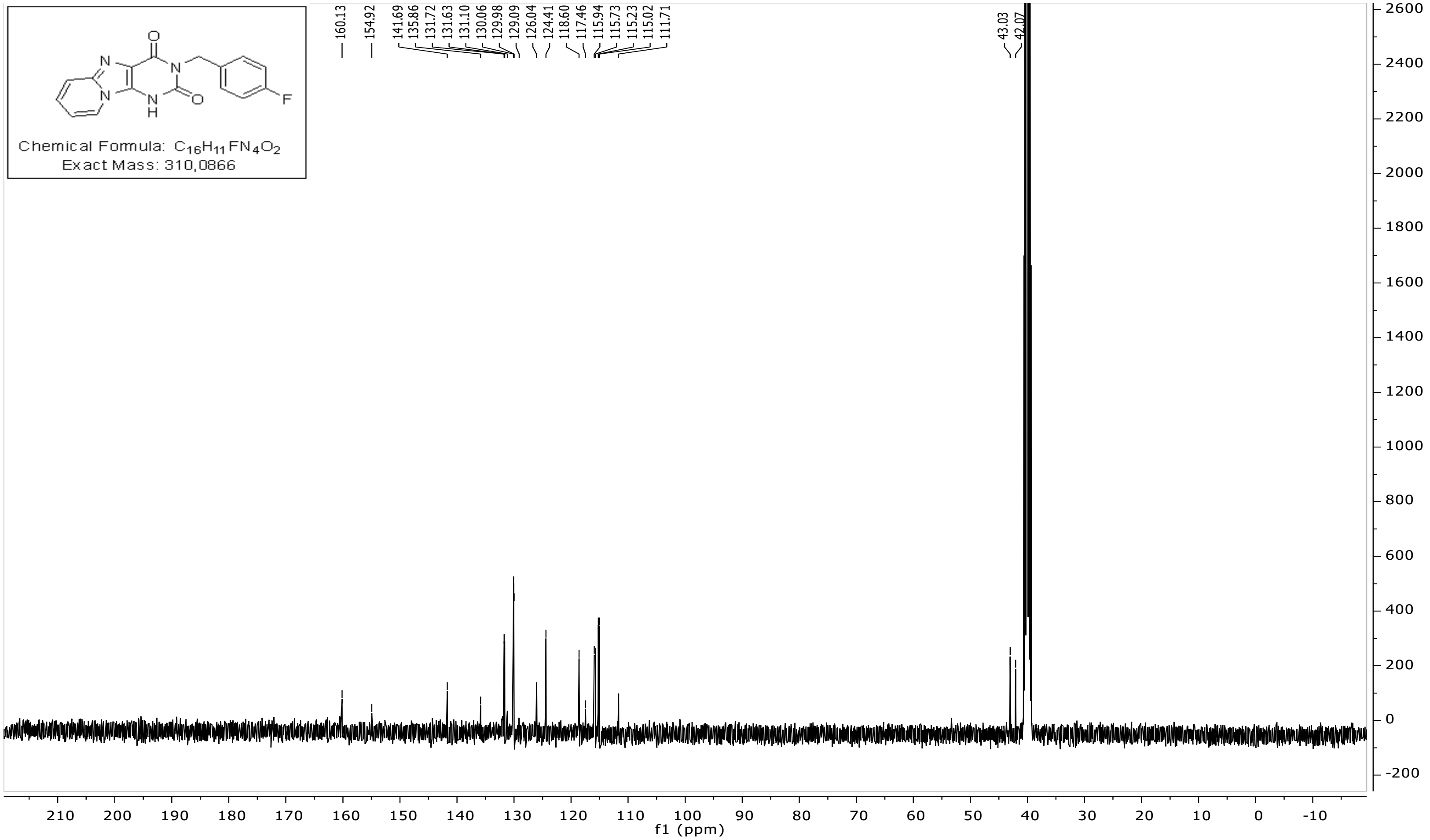

^{1}H NMR (DMSO-d6) of compound **10c**

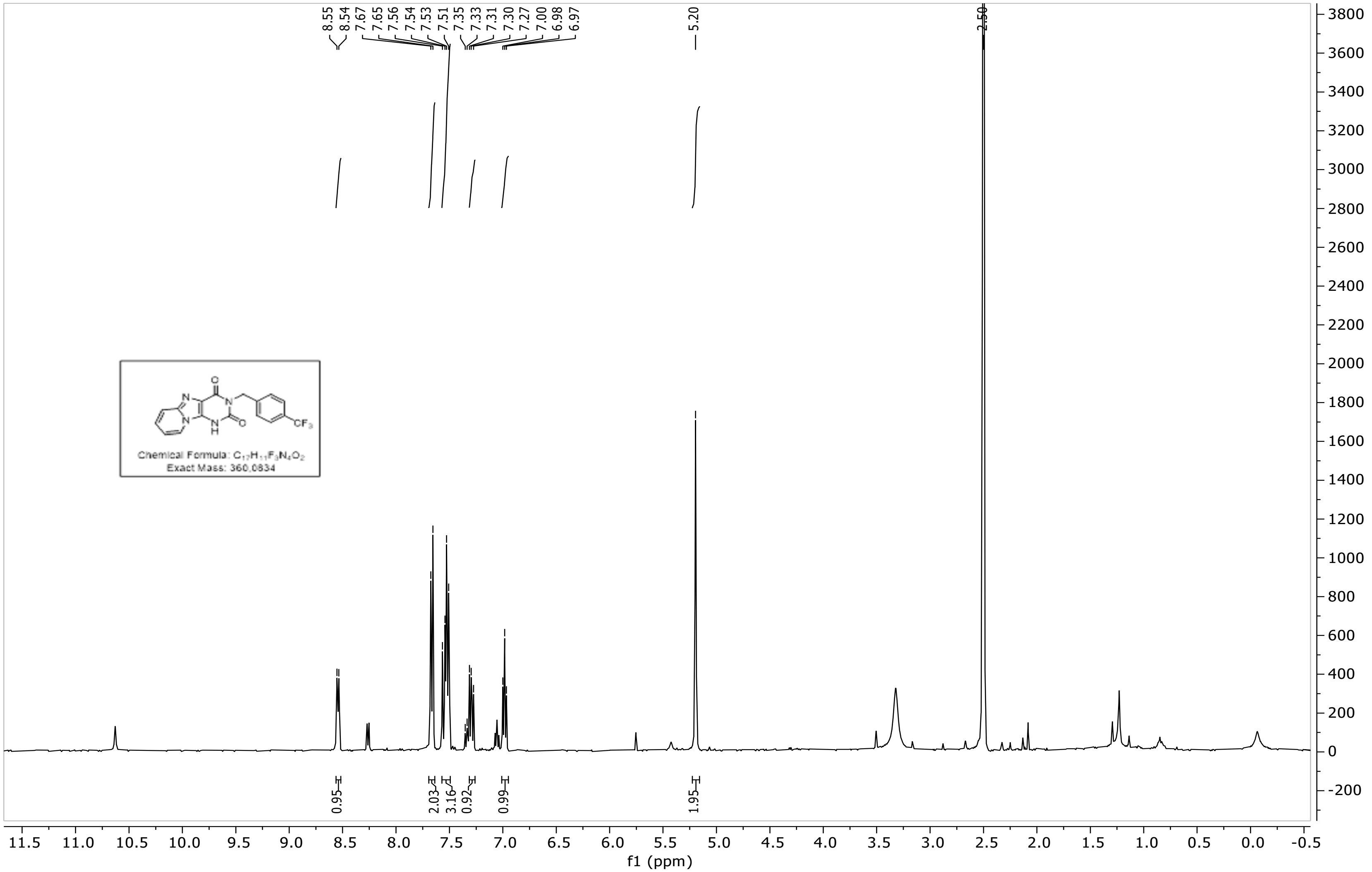

^{13}C NMR (DMSO-d6) of compound **10c**

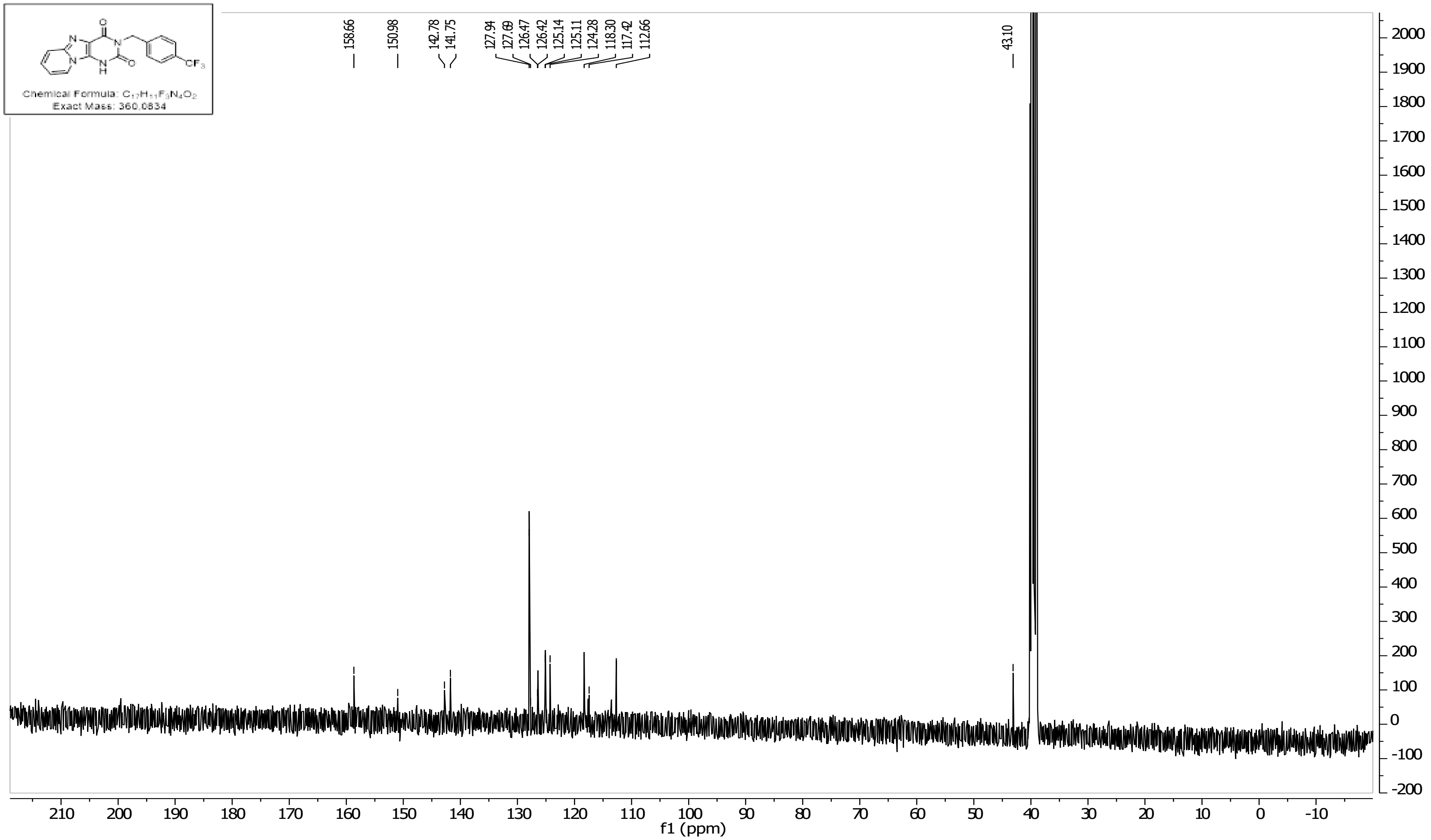

^{1}H NMR (DMSO-d6) of compound **10d**

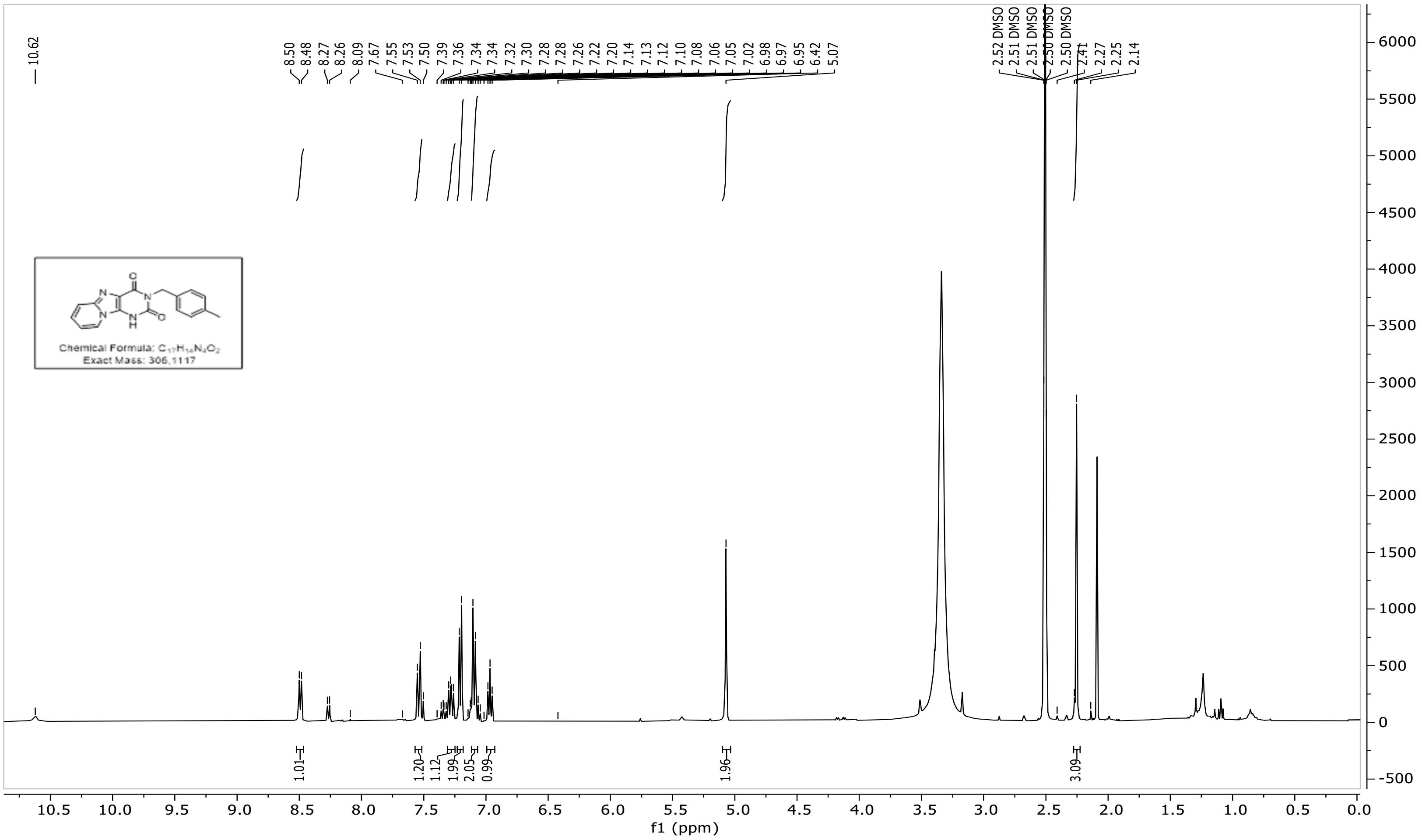

^{13}C NMR (DMSO-d6) of compound **10d**

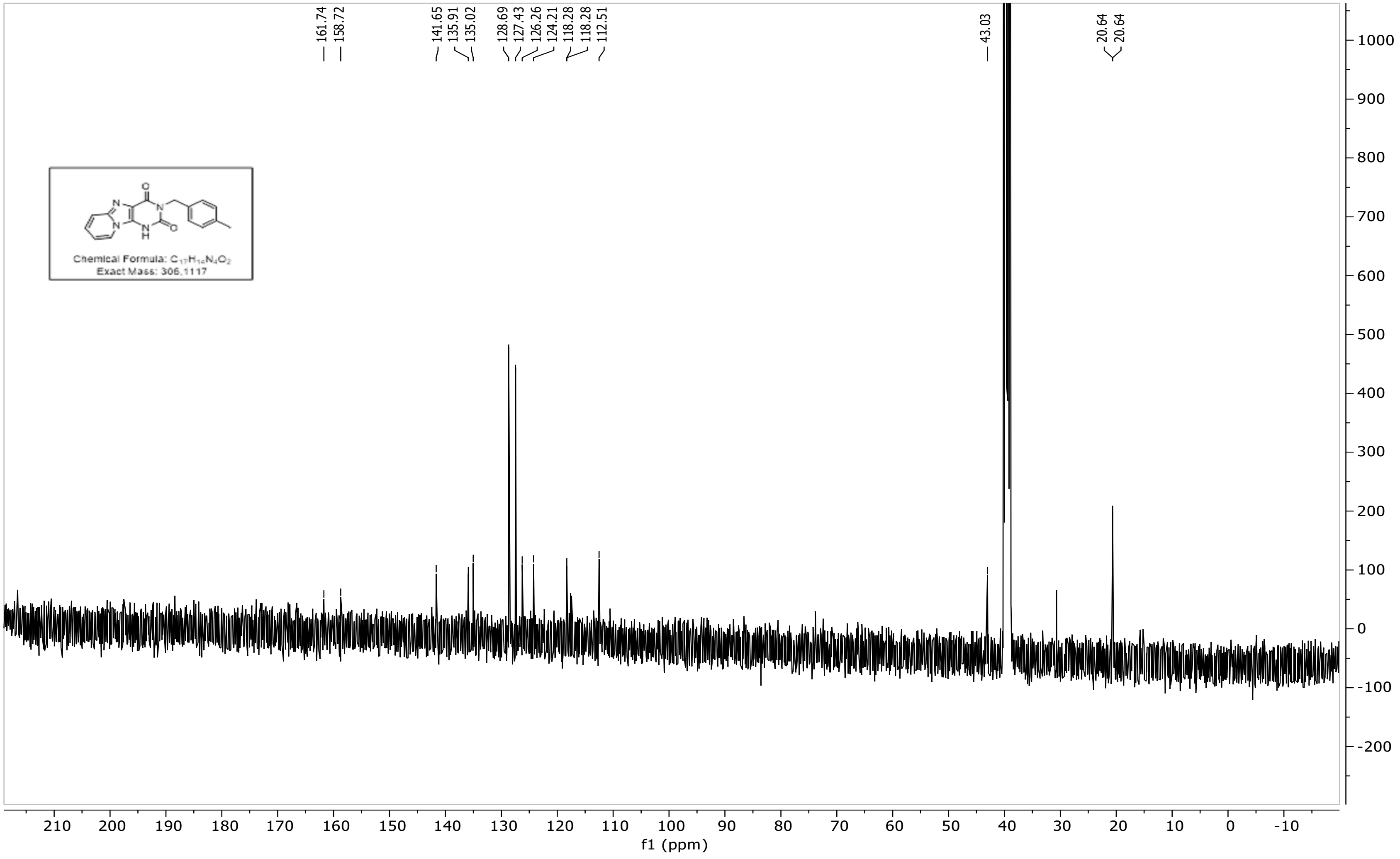

^{1}H NMR (DMSO-d6) of compound **13e**

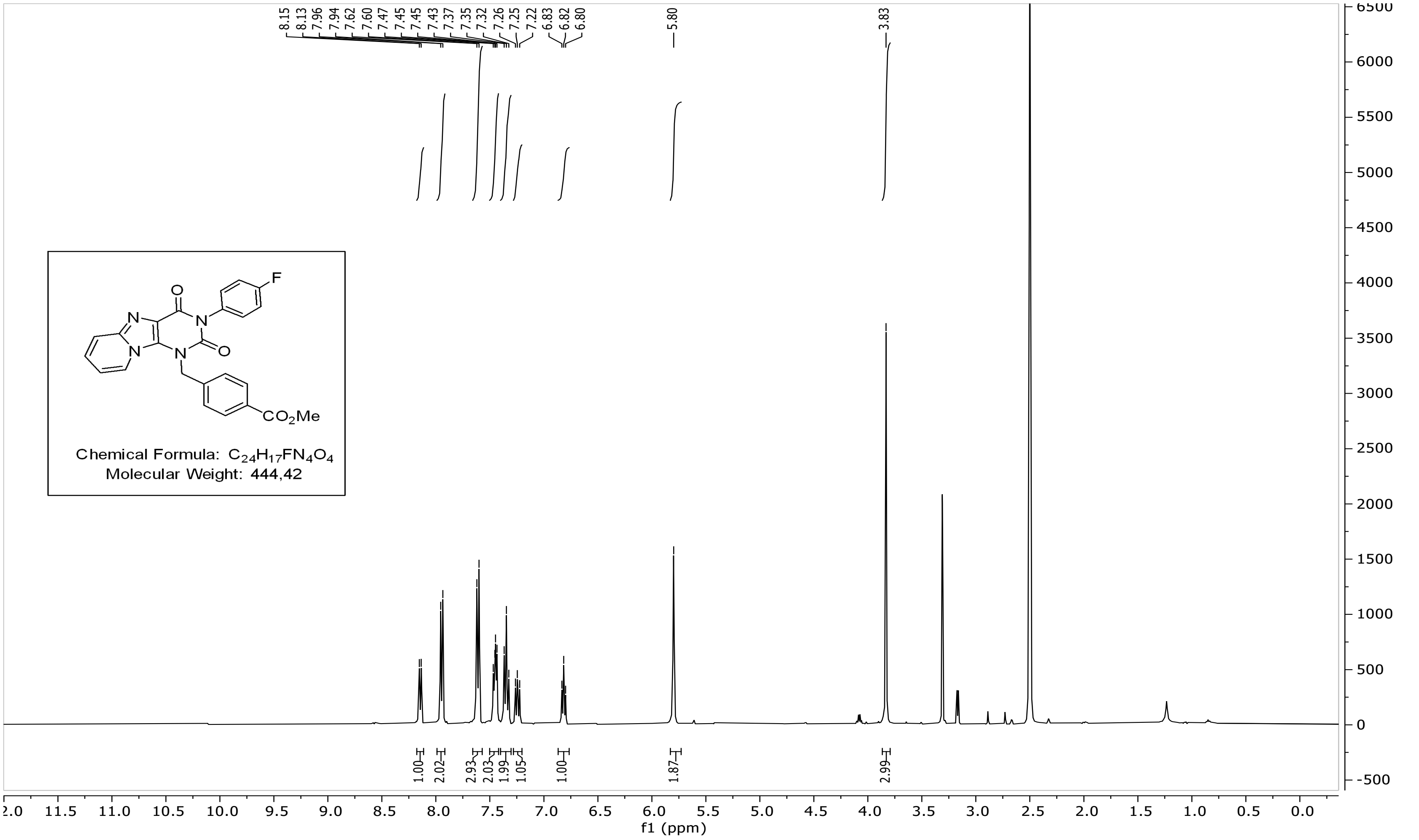

^{13}C NMR (DMSO-d6) of compound **13e**

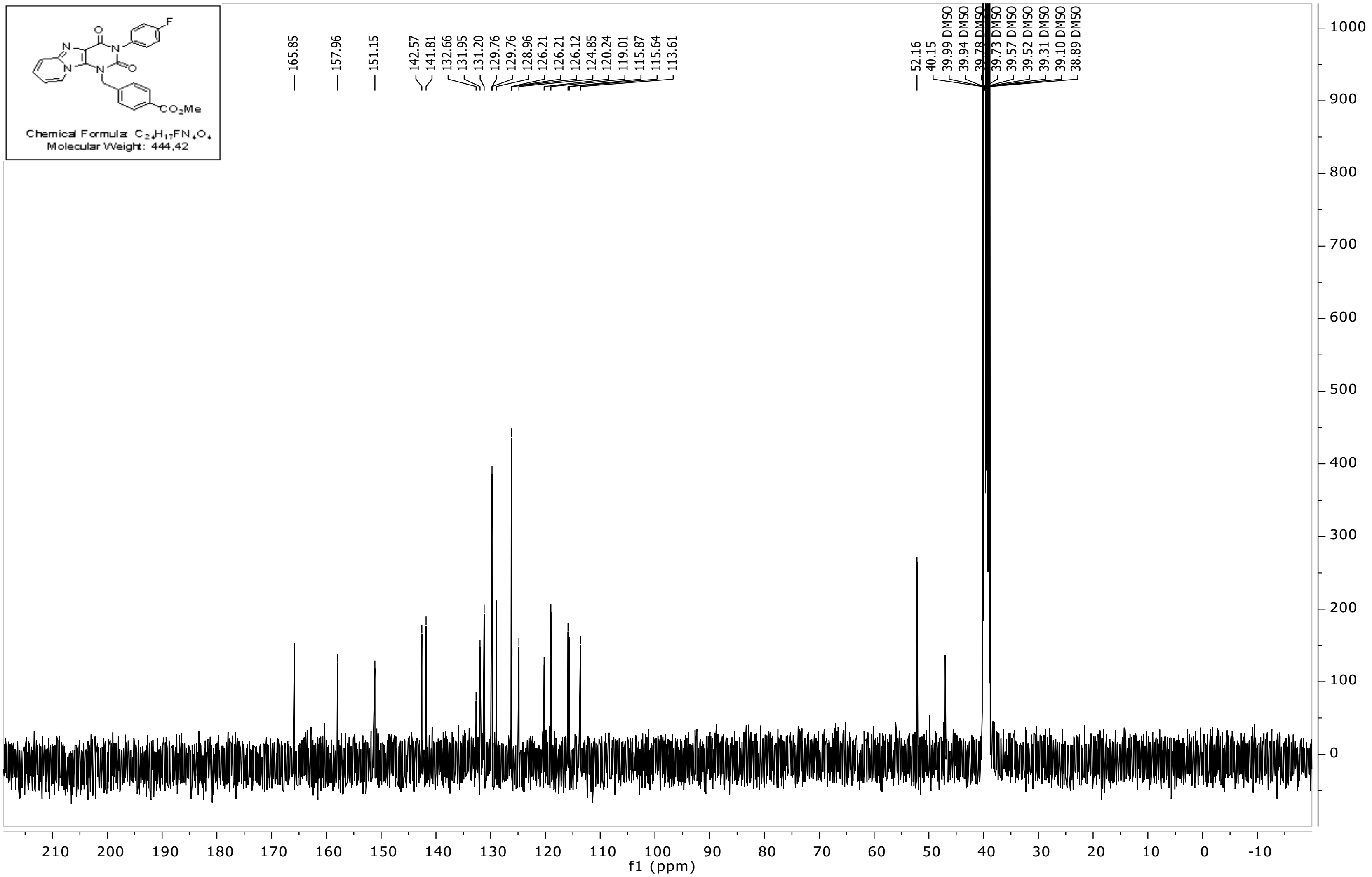

^{1}H NMR (DMSO-d6) of compound **18a**

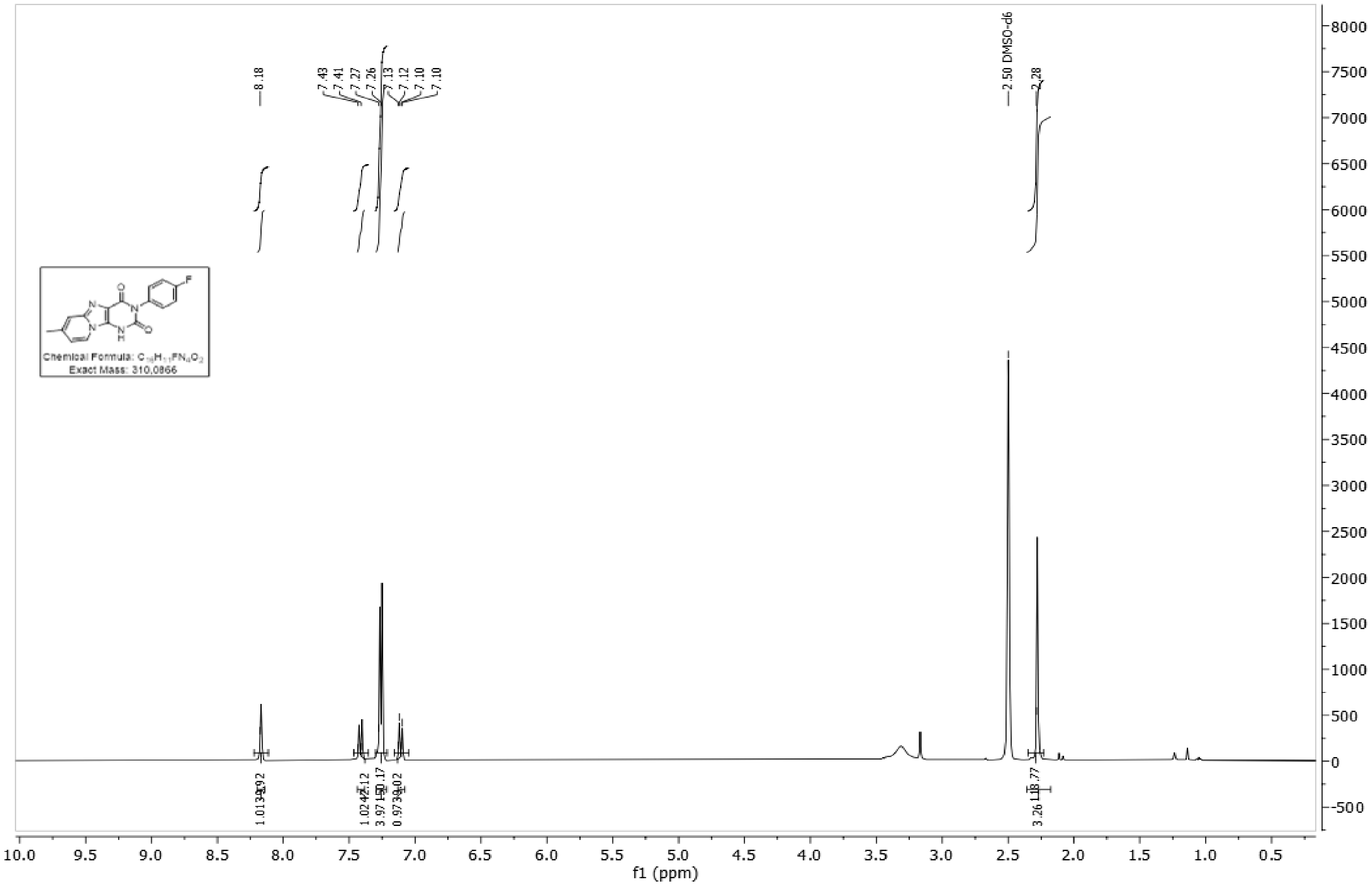

^{13}C NMR (DMSO-d6) of compound **18a**

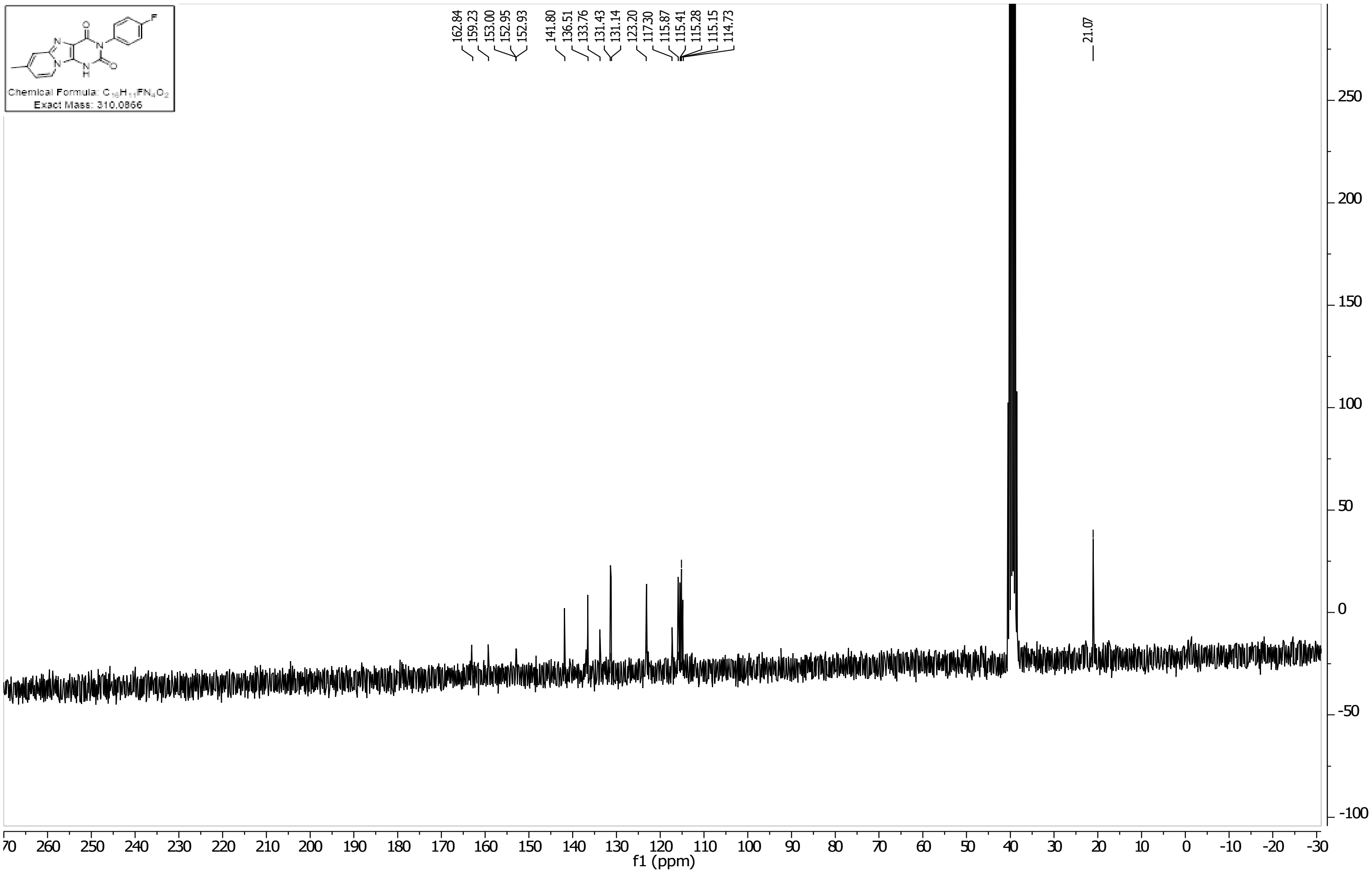

^{1}H NMR (DMSO-d6) of compound **20e**

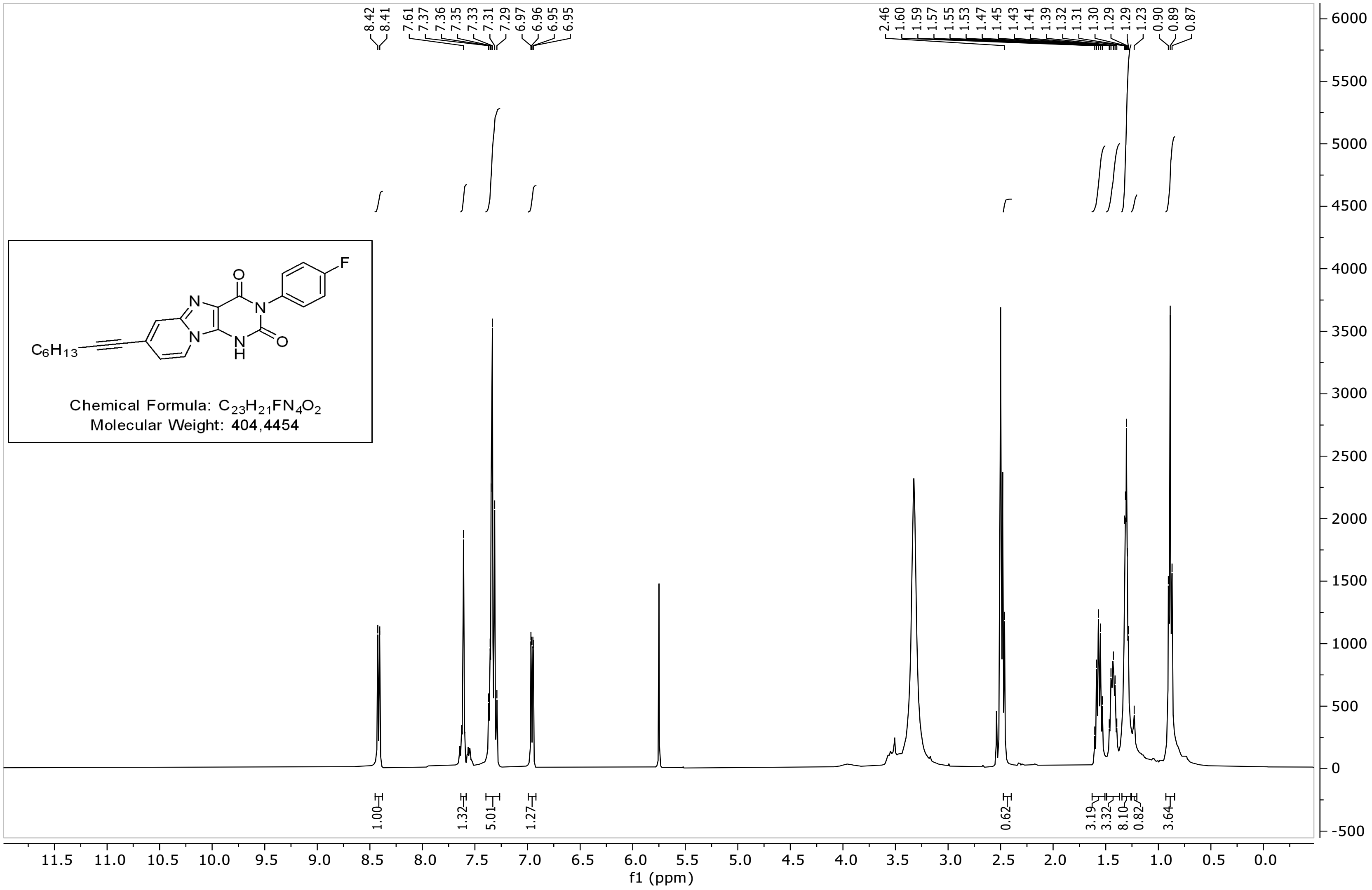

^{13}C NMR (DMSO-d6) of compound **20e**

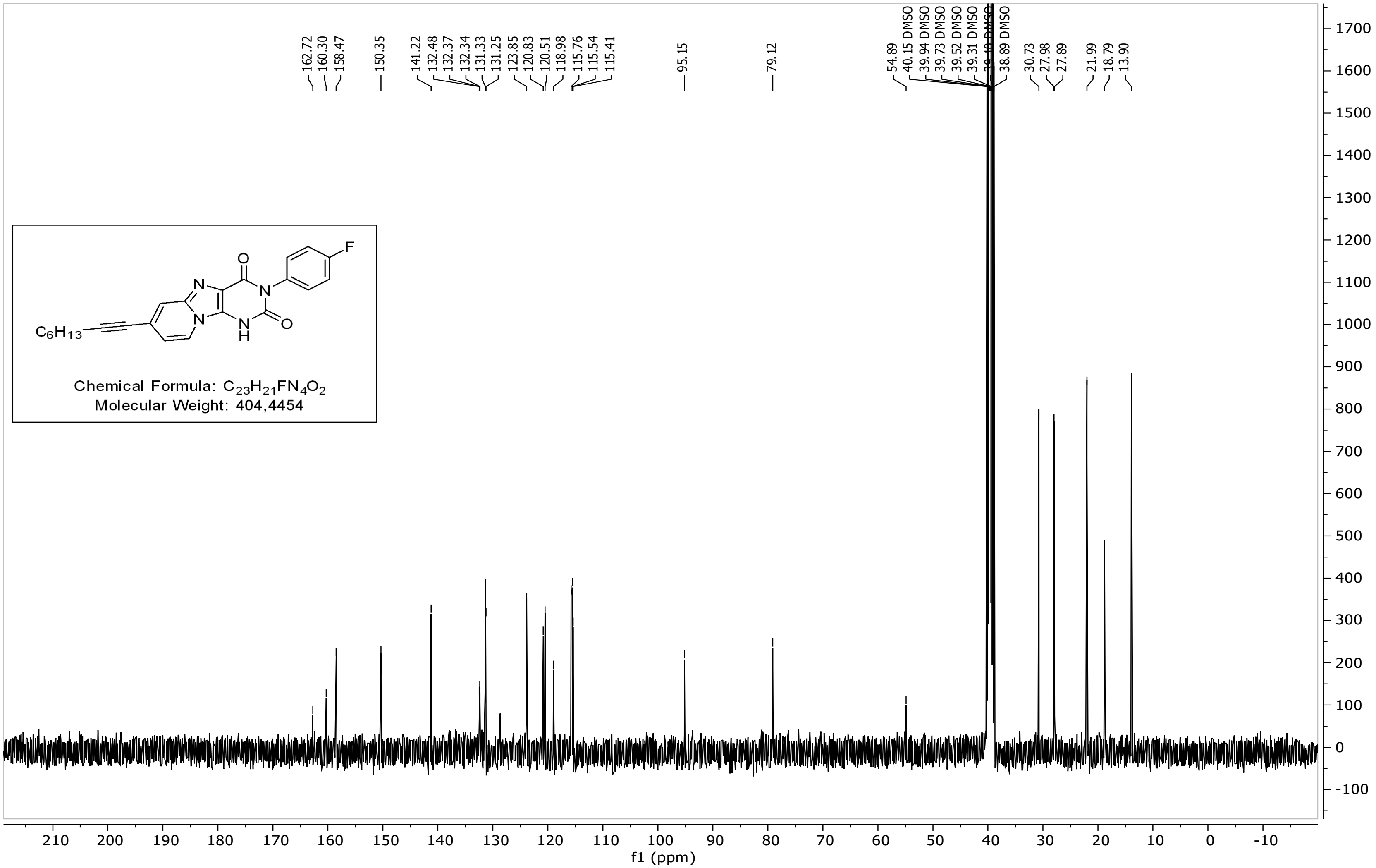

^{1}H NMR (DMSO-d6) of compound **21a**

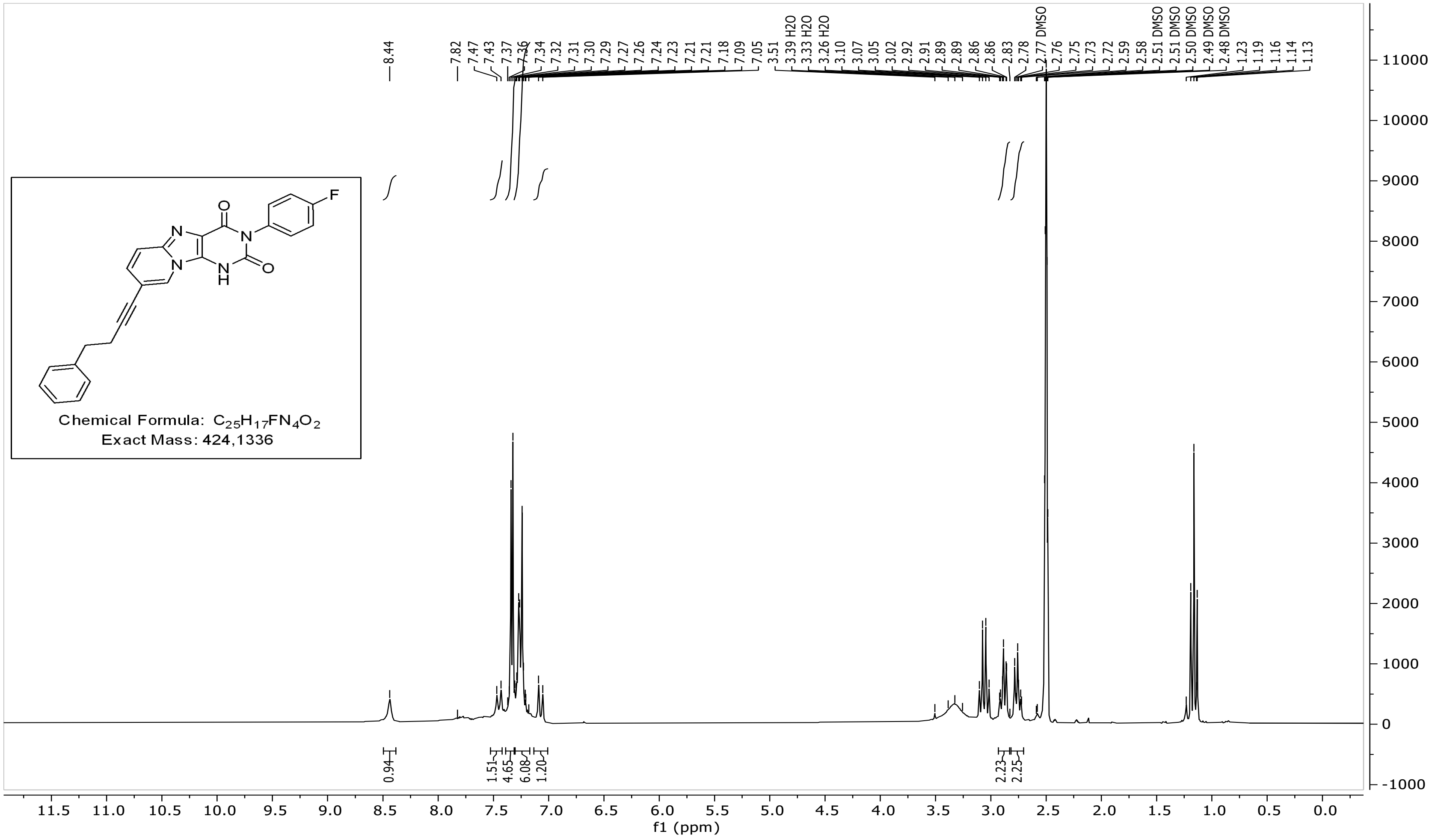

^{13}C NMR (DMSO-d6) of compound **21a**

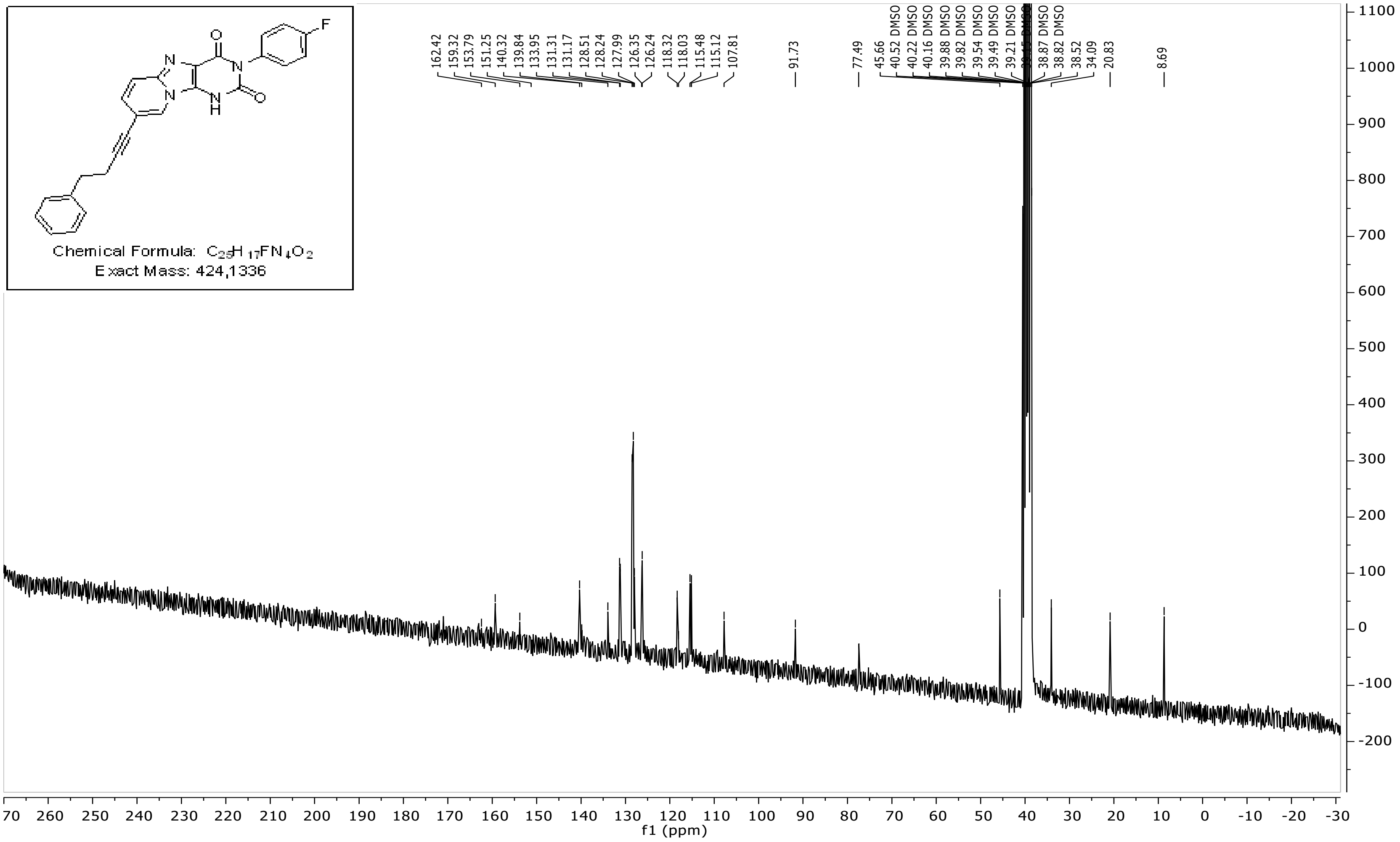

^{1}H NMR (DMSO-d6) of compound **22b**

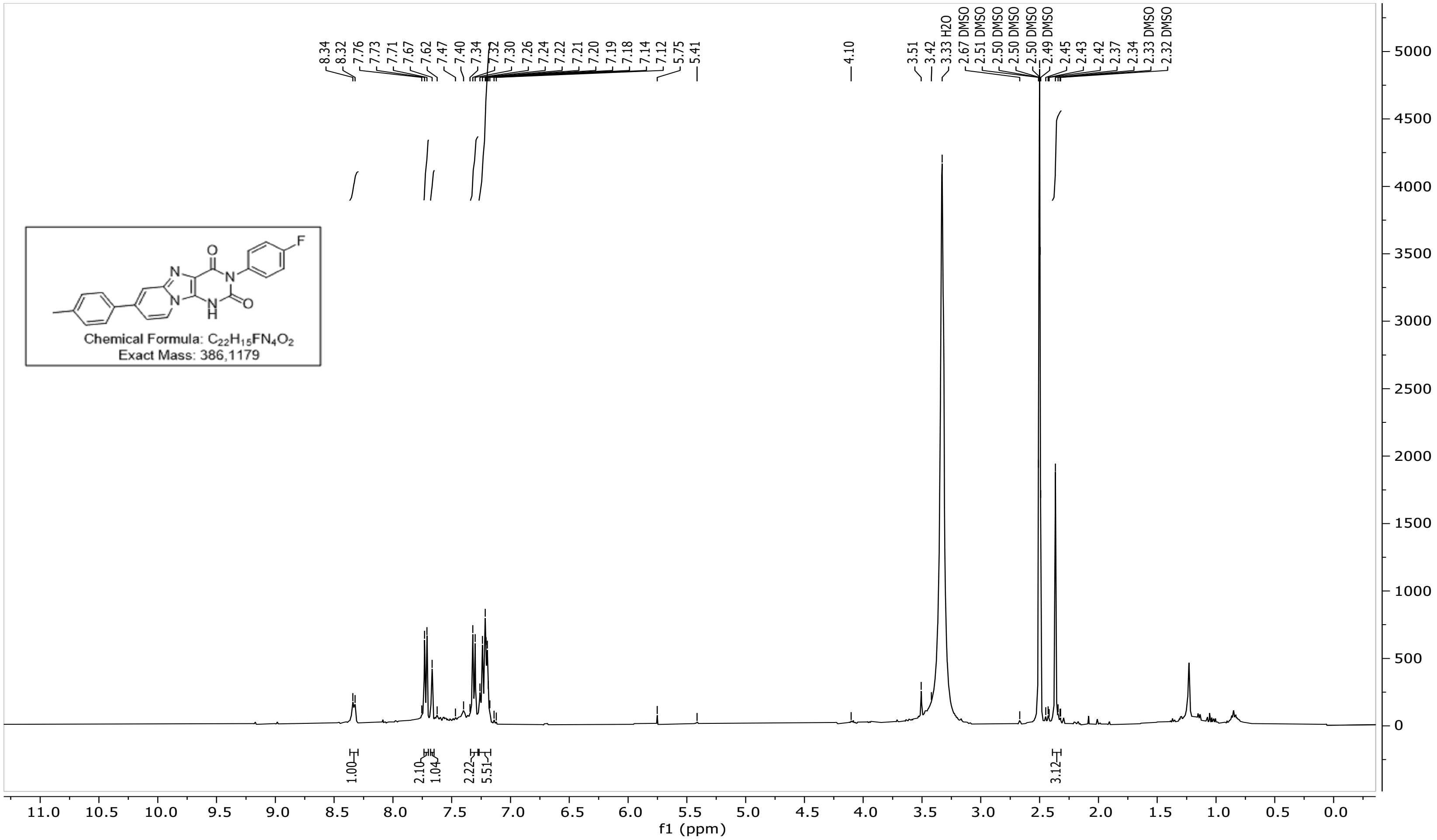

^{13}C NMR (DMSO-d6) of compound **22b**

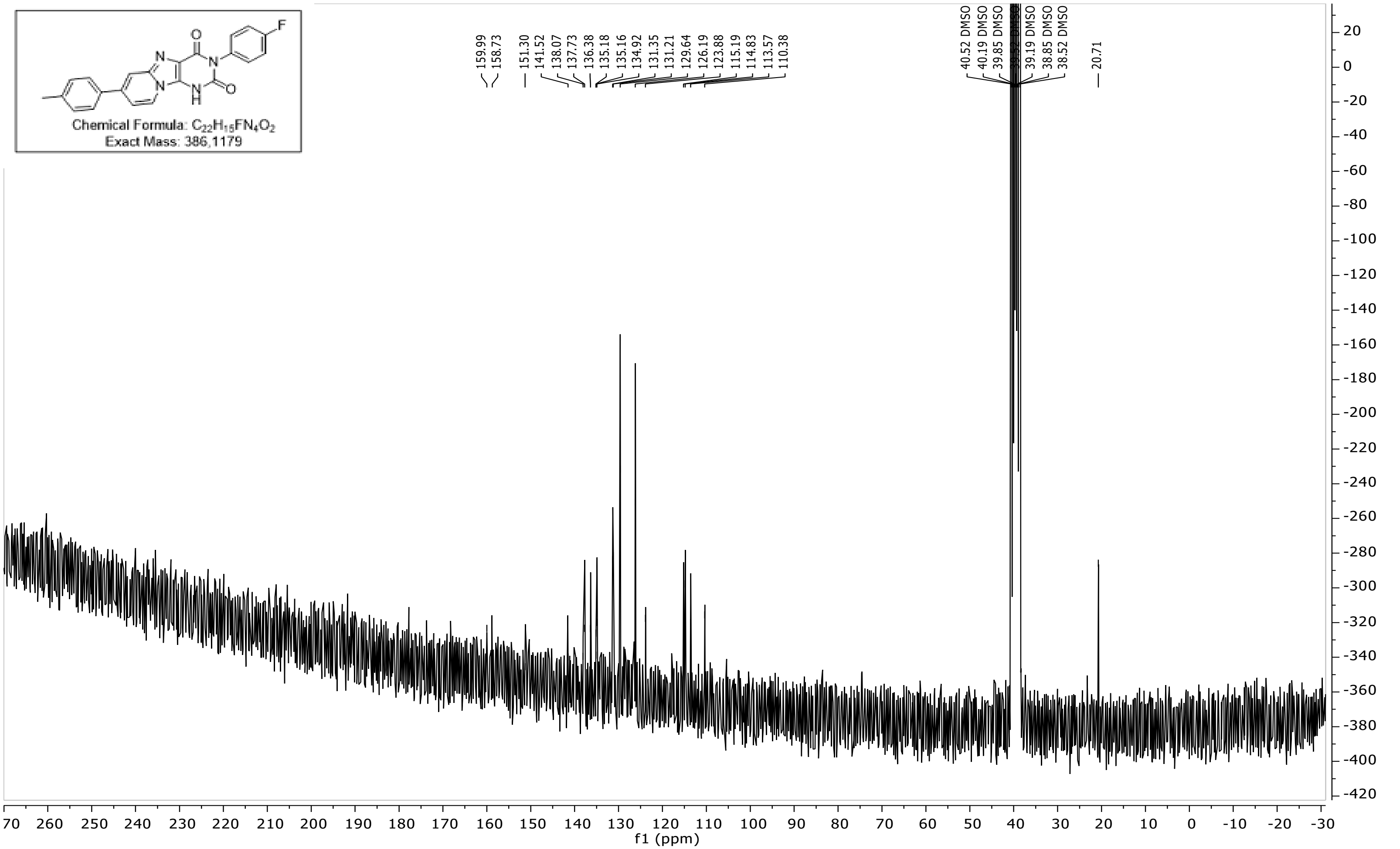

^{1}H NMR (DMSO-d6) of compound **23a**

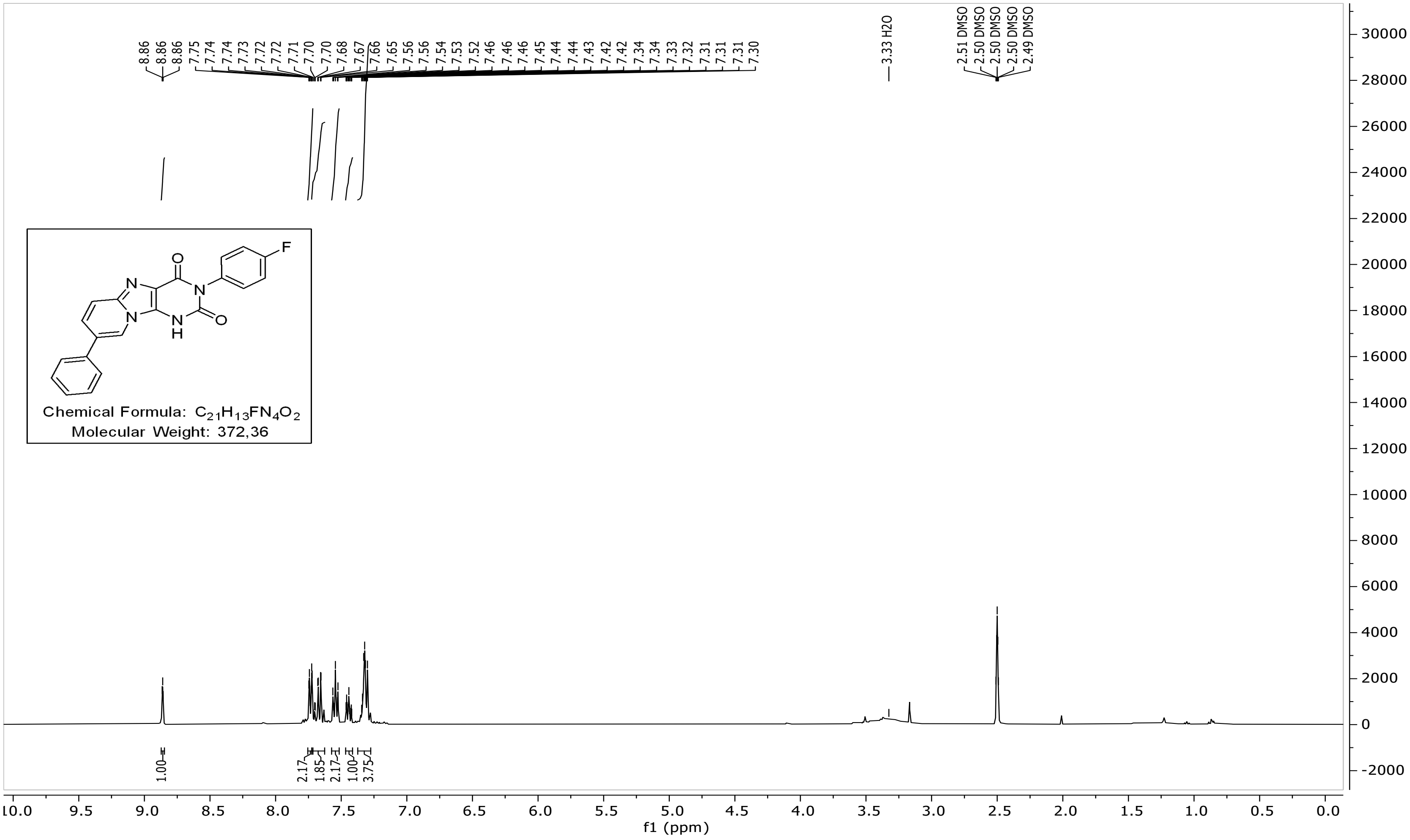

^{13}C NMR (DMSO-d6) of compound **23a**

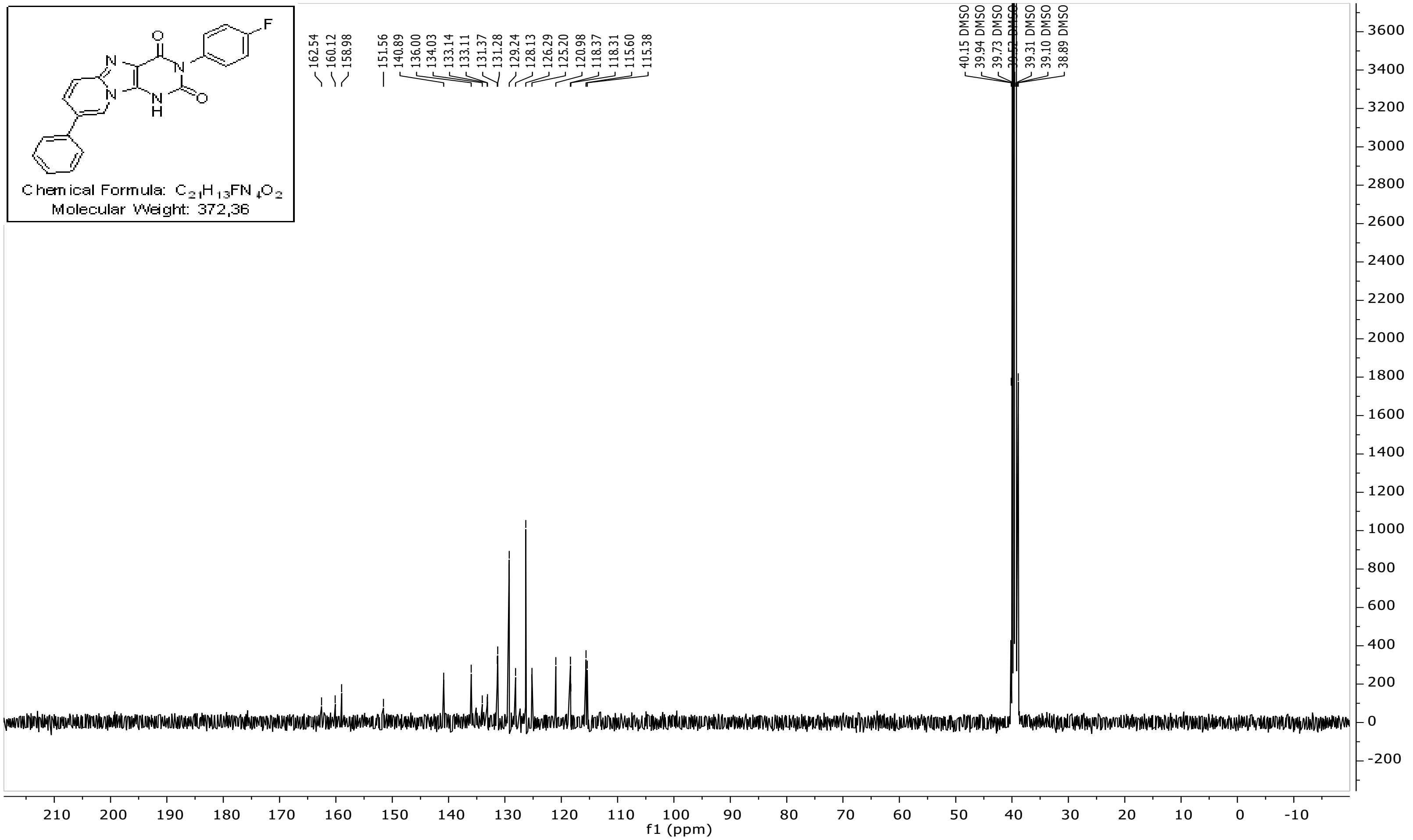

^{1}H NMR (DMSO-d6) of compound **23b**

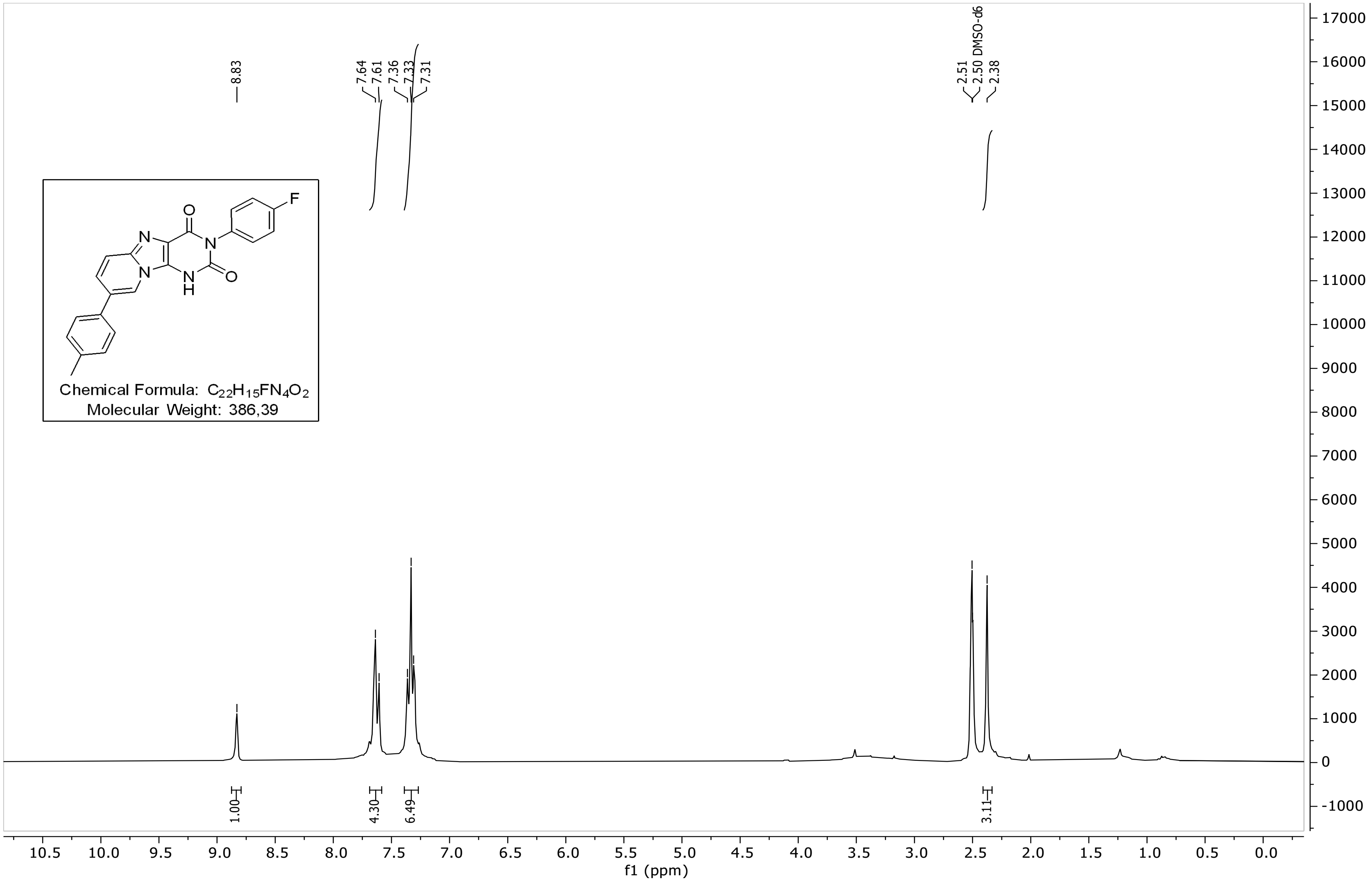

^{13}C NMR (DMSO-d6) of compound **23b**

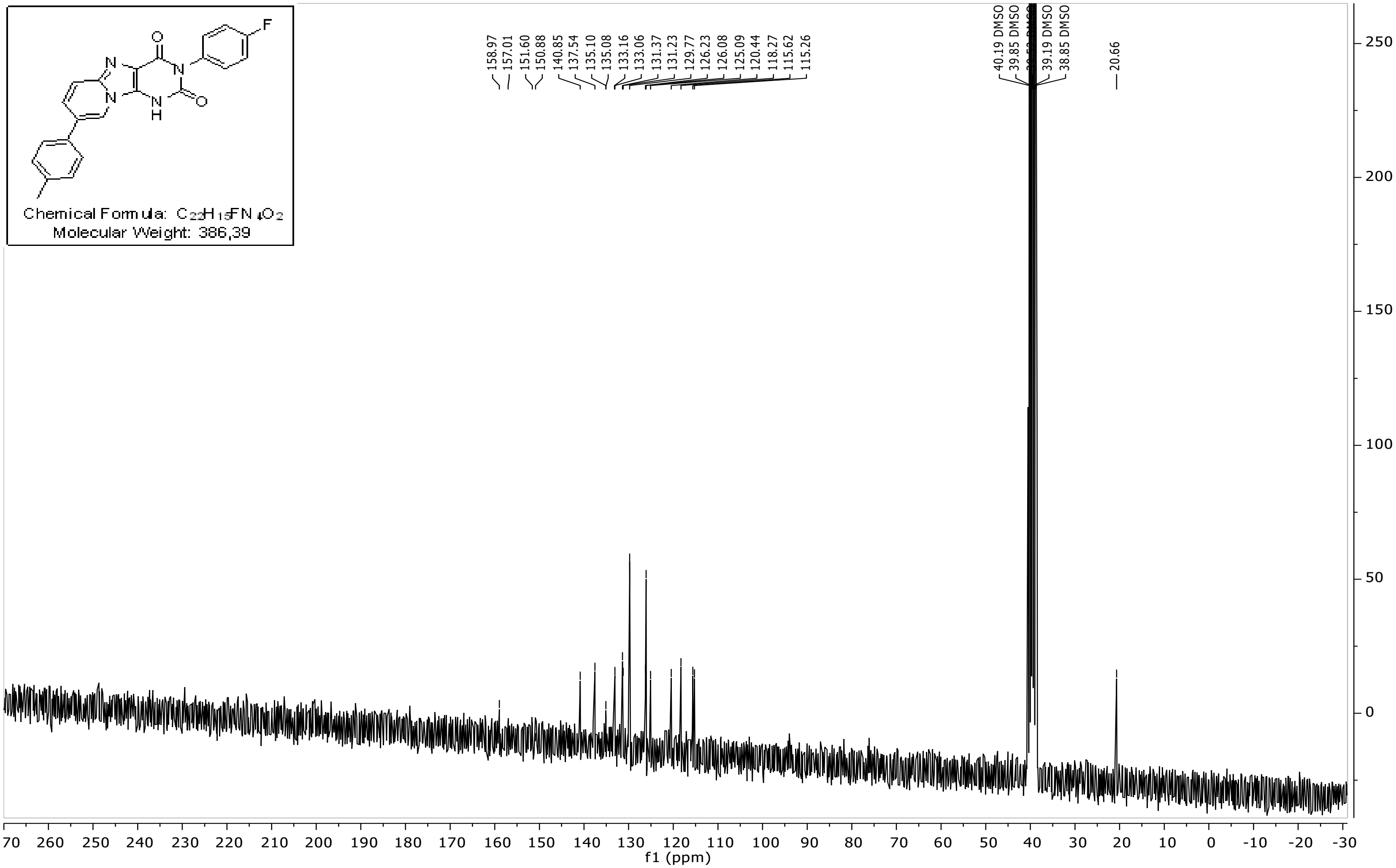

^{1}H NMR (DMSO-d6) of compound **23c**

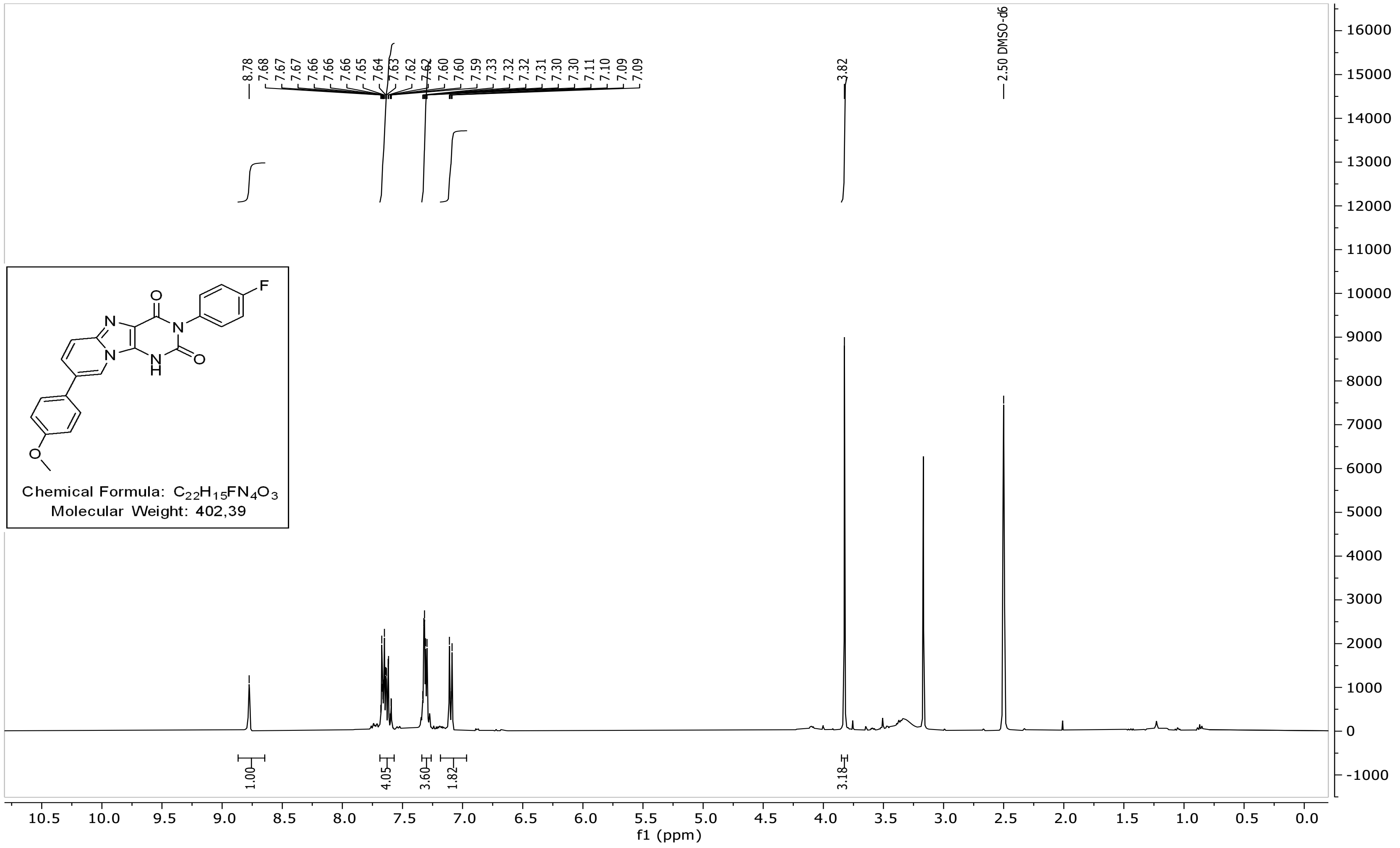

^{13}C NMR (DMSO-d6) of compound **23c**

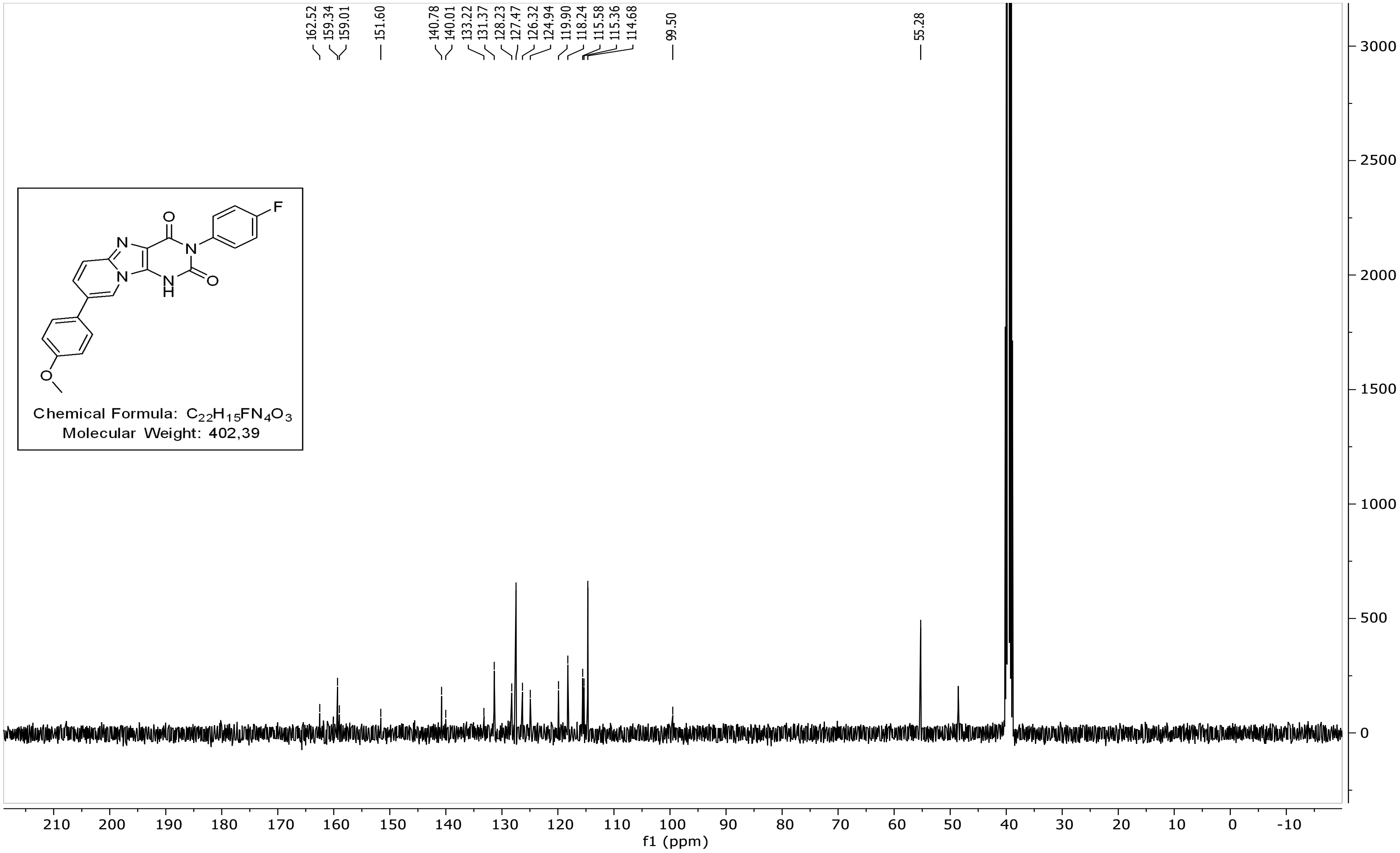

^{1}H NMR (DMSO-d6) of compound **23e**

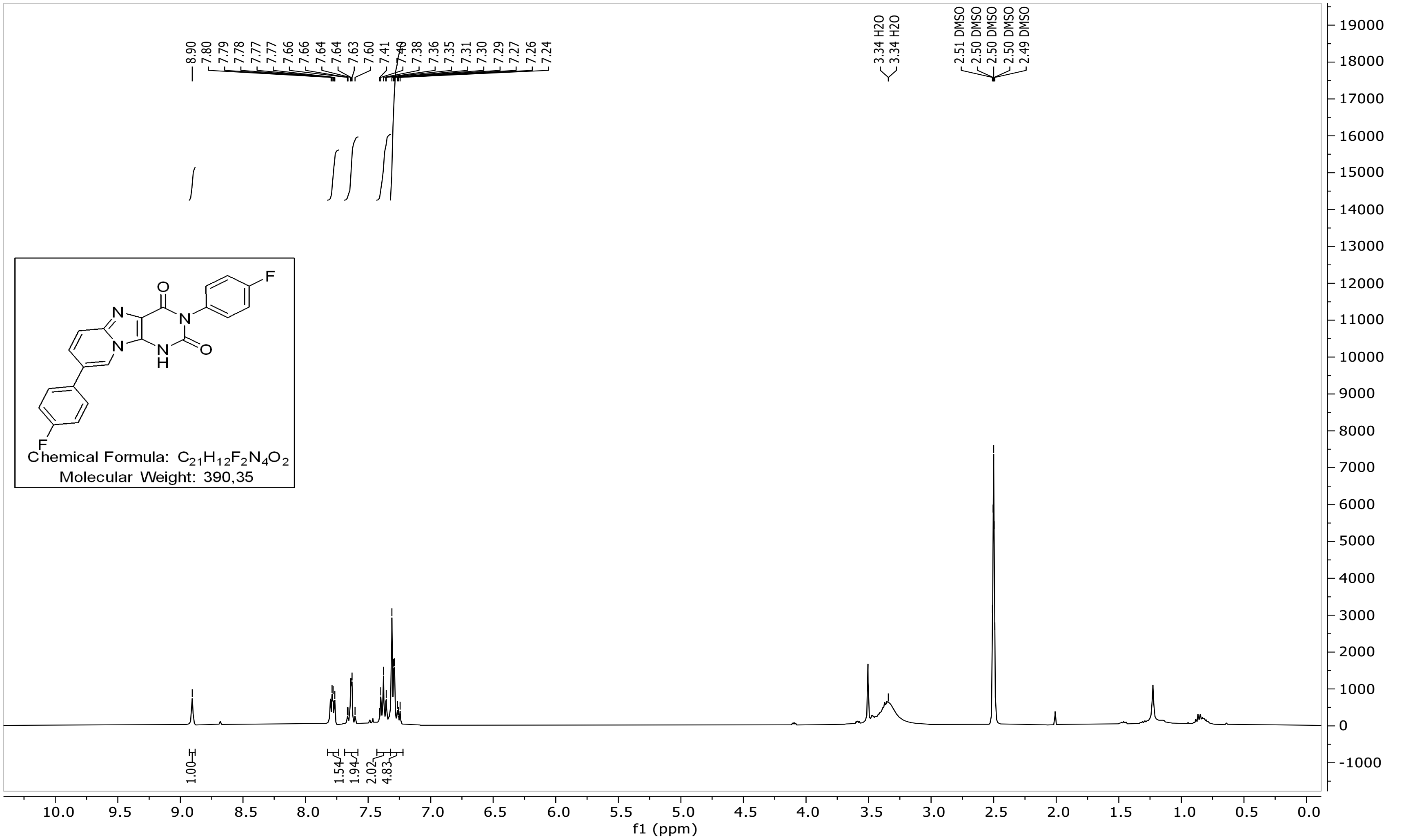

^{13}C NMR (DMSO-d6) of compound **23e**

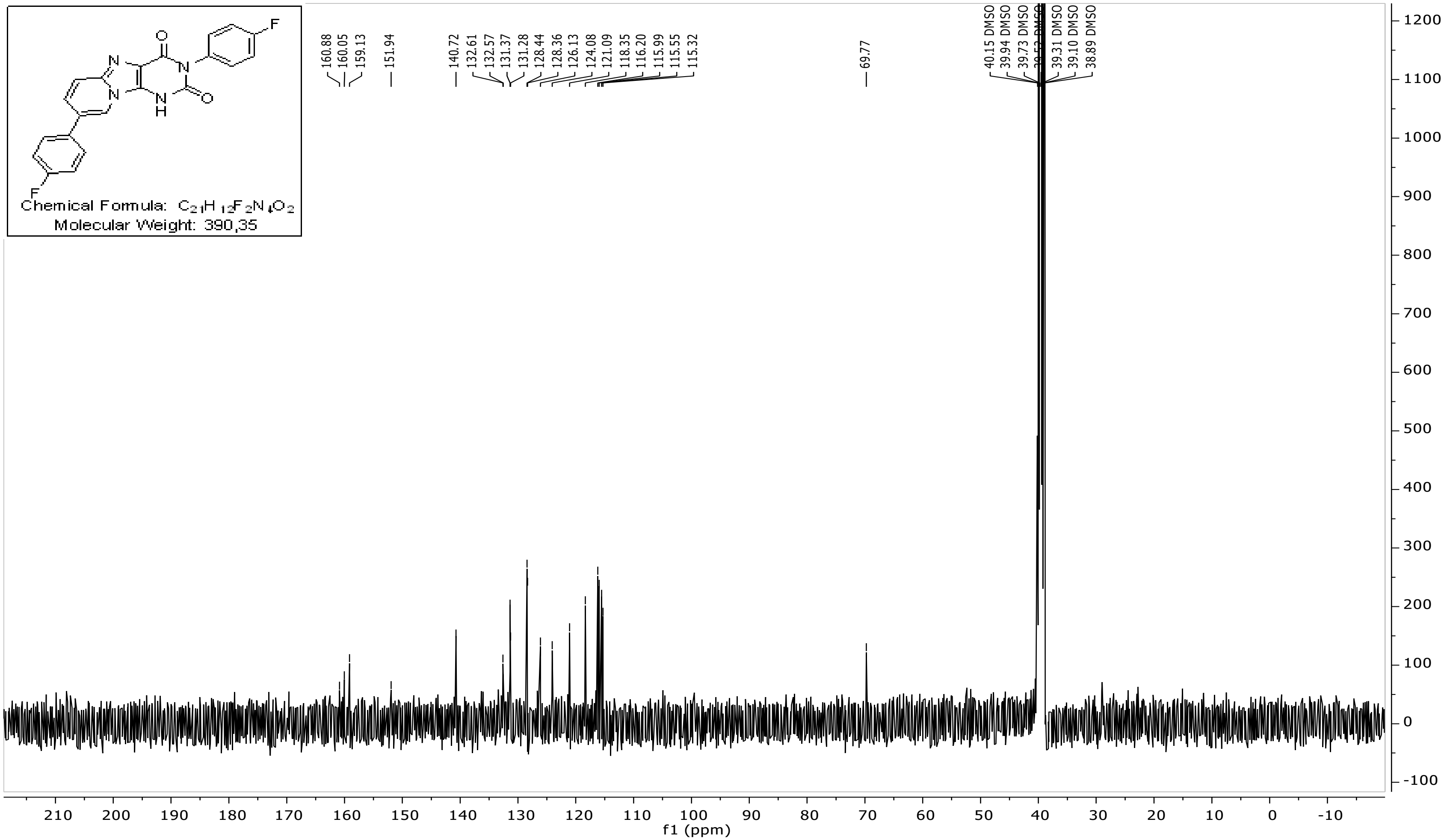

Supplement: Supplementary file 1 [file molecules-27-06216-s001.zip › molecules-1929618-supplementary.pdf]
